# Osteopontin promotes age-related adipose tissue remodeling through senescence-associated macrophage dysfunction

**DOI:** 10.1172/jci.insight.145811

**Published:** 2023-04-24

**Authors:** Daigo Sawaki, Yanyan Zhang, Amel Mohamadi, Maria Pini, Zaineb Mezdari, Larissa Lipskaia, Suzain Naushad, Lucille Lamendour, Dogus Murat Altintas, Marielle Breau, Hao Liang, Maissa Halfaoui, Thaïs Delmont, Mathieu Surenaud, Déborah Rousseau, Takehiko Yoshimitsu, Fawzia Louache, Serge Adnot, Corneliu Henegar, Philippe Gual, Gabor Czibik, Geneviève Derumeaux

**Affiliations:** 1 INSERM U955, Université Paris-Est Créteil, Créteil, France.; 2 AP-HP Vaccine Research Institute, Créteil, France.; 3 Université Côte d’Azur, INSERM U1065, C3M, Nice, France.; 4 Laboratory of Synthetic Organic and Medicinal Chemistry, Graduate School of Medicine, Dentistry, and Pharmaceutical Sciences, Okayama University, Okayama, Japan.; 5 Université Paris-Saclay, Inserm UMR-S-MD1197, Hôpital Paul Brousse, Villejuif, France.; 6 Gustave Roussy Cancer Center, Villejuif, France.; 7 AP-HP, Department of Physiology, Henri Mondor Hospital, FHU SENEC, Créteil, France.

**Keywords:** Aging, Immunology, Adipose tissue, Extracellular matrix, Macrophages

## Abstract

Adipose tissue macrophages (ATMs) play an important role in obesity and inflammation, and they accumulate in adipose tissue (AT) with aging. Furthermore, increased ATM senescence has been shown in obesity-related AT remodeling and dysfunction. However, ATM senescence and its role are unclear in age-related AT dysfunction. Here, we show that ATMs (a) acquire a senescence-like phenotype during chronological aging; (b) display a global decline of basic macrophage functions such as efferocytosis, an essential process to preserve AT homeostasis by clearing dysfunctional or apoptotic cells; and (c) promote AT remodeling and dysfunction. Importantly, we uncover a major role for the age-associated accumulation of osteopontin (OPN) in these processes in visceral AT. Consistently, loss or pharmacologic inhibition of OPN and bone marrow transplantation of OPN^–/–^ mice attenuate the ATM senescence-like phenotype, preserve efferocytosis, and finally restore healthy AT homeostasis in the context of aging. Collectively, our findings implicate pharmacologic OPN inhibition as a viable treatment modality to counter ATM senescence-mediated AT remodeling and dysfunction during aging.

## Graphical abstract

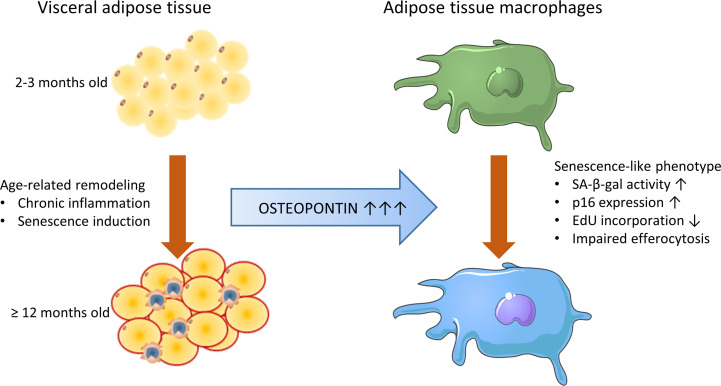

## Introduction

In obesity, adipose tissue (AT) undergoes cellular senescence involving activation of adipose tissue macrophages (ATMs), which enhances AT remodeling through proinflammatory and profibrotic signaling (1–5). Macrophages play a pivotal role in both the induction and resolution of inflammation that results in cellular dysfunction and AT damage. Notably, increased infiltration of proinflammatory macrophages in AT impairs the secreted adipokine profile and contributes to insulin resistance through a senescence-associated secretory phenotype (SASP) (6–8). Indeed, ATMs are an important source of chemokines, matrix metalloproteinases, and other profibrotic and inflammatory mediators that collectively constitute the SASP. Macrophages are also critically involved in AT remodeling because they are key to the clearance of obesity-associated senescent or damaged AT cells (9, 10). Recent observations suggest that senescence initiates tissue remodeling by recruiting immune cells through SASP to allow clearance and regeneration of the damaged tissue (9, 11). With persistent damage, as occurs in obesity and aging, however, clearance and regeneration may be compromised by senescence-associated macrophage dysfunction (9).

As in obesity (12), AT remodeling has been described during aging (13). Indeed, aging increases ectopic and visceral AT (VAT) depots (14). Age-associated VAT remodeling includes adipocyte hypertrophy, deposition of extracellular matrix, and accumulation of immune cells (13). The role of ATMs, in aggravating AT remodeling, and in particular their senescence, remain an open question in aging (15). Specifically, it is still unclear whether ATMs become senescent per se or by exposure to accumulation of senescent AT cells (16–18). Initially, expression of p16INK4a and senescence-associated β-galactosidase (SA-β-Gal) was reported in VAT of aged mice (16). Later the same group demonstrated that the expression of these markers was reversible with immunomodulatory stimuli (17). Their subsequent studies of old p16-tdTom reporter mice established that cells displaying strong p16INK4a promoter activity show features of senescence, such as reduced proliferation, transcriptional upregulation of SASP factors, and increased SA-β-Gal activity (18). Because of the lack of reliable markers that unequivocally identify senescent cells in vivo, we need to combine multiple hallmarks of cellular senescence in addition to evidence of dysfunction, such as impaired efferocytosis in ATMs. Thus, exploring whether ATMs become senescent during aging requires further investigation to understand underlying mechanisms that may lead to the development of innovative treatment.

Interestingly, we have recently established VAT as the major source of osteopontin (OPN) during aging (19). OPN is a matricellular protein involved in intracellular and extracellular signaling mediating cell-to-cell interactions, immune cell function, inflammation, and tissue remodeling (20, 21). Beyond the reported role of OPN in AT proinflammatory status (21–23), we identified OPN as an important SASP component (19, 24) among a variety of growth factors, proinflammatory cytokines/chemokines, and adipokines, with a major role in inducing remote cardiac tissue remodeling by modulating fibroblast function (19, 24). However, the link among age-related OPN production, VAT senescence, and impairment of ATM function is elusive.

Here we show that during chronological aging ATMs acquire several features of senescent cells, which impair ATM function, and contribute to age-related VAT remodeling and dysfunction, a process mediated by OPN. Our findings highlight OPN inhibition as a potential therapeutic intervention to rejuvenate VAT, thus promoting healthy aging.

## Results

### Aging promotes accumulation of senescent cells in VAT.

**Figure 1:**
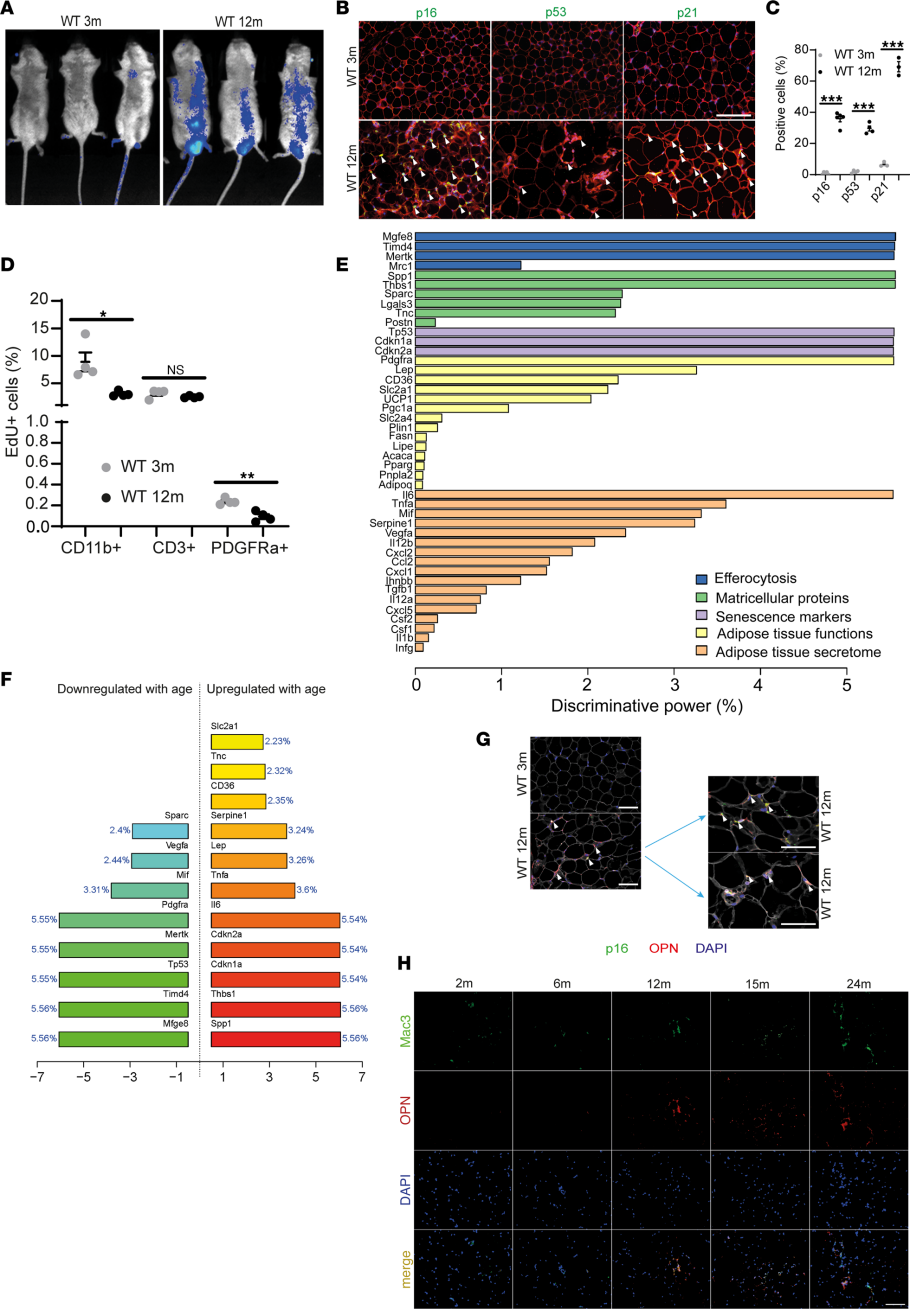
Aging promotes accumulation of senescent cells in visceral adipose tissue. (**A**) Representative luminescent images from 3- and 12-month-old p16-luciferase mice. (**B**) Representative immunostaining of VAT (p16/p53/p21, green; wheat germ agglutinin (WGA), red; DAPI, blue), arrowheads indicate green-positive cells; scale bar: 50 μm. (**C**) Quantification of positive cells (green) in the percentage of total cell number (DAPI, p16, *n* = 5 mice/group; p21, *n* = 4 mice/group; p53, *n* = 3 mice/group). (**D**) EdU^+^ cells in VAT-derived CD11b^+^ macrophages, CD3^+^ lymphocytes, and PDGFRa^+^ preadipocytes of 3- and 12-month-old WT mice (*n* = 4/group). (**E**) Discriminative power of qRT-PCR results for transcript levels of interest. Grouped in 5 ad hoc categories in relation to aging and presented in decreasing order of the most discriminant parameter in each category. (**F**) The most informative gene expression parameters in relation to aging, and the direction (i.e., positive or negative) of their association with aging. (**G**) Representative immunofluorescence of VAT from 3- and 12-month-old WT mice (p16, green; OPN, red; WGA, white; DAPI, blue); arrowheads indicate p16^+^ cells; scale bar: 50 μm (selected from *n* = 3/condition). (**H**) Representative images of OPN/Mac3 (macrophages marker) stained VAT derived from WT mice aged between 2 and 24 months as indicated; scale bar: 50 μm (selected from *n* = 3/condition). Data are presented as original images (**A**, **B**, **G**, and **H**), individual values with mean ± SEM and analyzed with 2-tailed unpaired Student’s *t* test (**C** and **D**) or discriminative power analysis as described in the Methods (**E** and **F**); ns, nonsignificant, *P < 0.05, **P < 0.01, ***P < 0.001.

Because age-related accumulation of strongly p16^+^ senescent cells has been reported in AT (18), we assessed p16-luciferase activity in the zone of epididymal VAT in 3- vs. 12-month-old mice by using a hemizygous luciferase p16-knockin mouse model (p16-Luc^+/–^), which faithfully reports expression of the senescence biomarker p16 (25). In line with previous findings (25, 26), we detected higher luciferase signal in 12- compared with 3-month-old p16-Luc^+/–^ mice (Figure 1A) and an induction of other classic senescence biomarkers (by transcriptomic analysis: *Cdkna2* and *Cdkna1*; by staining: p16, p21, and p53; Figure 1, B and C, and Supplemental Figure 1, A and B; supplemental material available online with this article; https://doi.org/10.1172/jci.insight.145811DS1), together with SA-β-Gal activity (Supplemental Figure 1C) in VAT of C57BL/6JRj (WT) male mice at 12 months of age. Consistent with the reported increase in senescent-like ATMs (16–18), we detected an increase in senescence biomarker expression (p16, p21, H3K9me3) in ATMs starting at 12 months of age (Supplemental Figure 1, D–F), along with a reduced 5-ethynyl-2’-deoxyuridine (EdU) incorporation in both CD11b^+^ ATMs and PDGFRα^+^ preadipocytes by flow cytometry (Figure 1D). We did not detect a reduced proliferative capacity in CD3^+^ T lymphocytes (Figure 1D). Although we observed a comparable induction of some senescence markers (p21 and H3K9me3) in aged inguinal subcutaneous adipose tissue (SAT), intriguingly p16 was not induced there (Supplemental Figure 1, G–I).

Moreover, senescence-like changes in VAT associated with increased transcript levels of proinflammatory and matricellular proteins of SASP (Supplemental Figure 1, J and K) (24). One of the upregulated genes encoded OPN (*Spp1*) and was among the most discriminative genes associated with VAT aging, along with senescence-related (p21 and p16) and efferocytosis-related genes (Figure 1, E and F). Notably, OPN colocalized with p16 (Figure 1G), and its expression dynamics (Figure 1H) coincided with the induction of senescence biomarkers (Supplemental Figure 1, D–F) in VAT. In VAT of 12-month-old WT mice, OPN was expressed predominantly by ATMs and to a lesser extent by CD3^+^ T lymphocytes (Supplemental Figure 1, L and M). Interestingly, consistent with the lack of age-dependent p16 induction in SAT (Supplemental Figure 1I), OPN expression did not increase with age in SAT (Supplemental Figure 1N).

These age-related VAT changes paralleled with an increase in body and organ weight (including AT depots), adipocyte hypertrophy, disturbed glucose, and fatty acid metabolism–related gene expression, higher adipokine levels, and interstitial fibrosis of VAT, together with impaired glucose tolerance or insulin sensitivity (Supplemental Figure 1, O–W).

We conclude, following the current gold standard of senescence detection (i.e., high-level p16 activation, reduced replication or proliferation, and expression of senescence-associated transcripts, e.g., SASP), that age-dependent VAT senescence entails the accumulation of senescent-like ATMs that may be functionally related to increased OPN expression.

### OPN is a critical regulator of AT and macrophage senescence.

**Figure 2:**
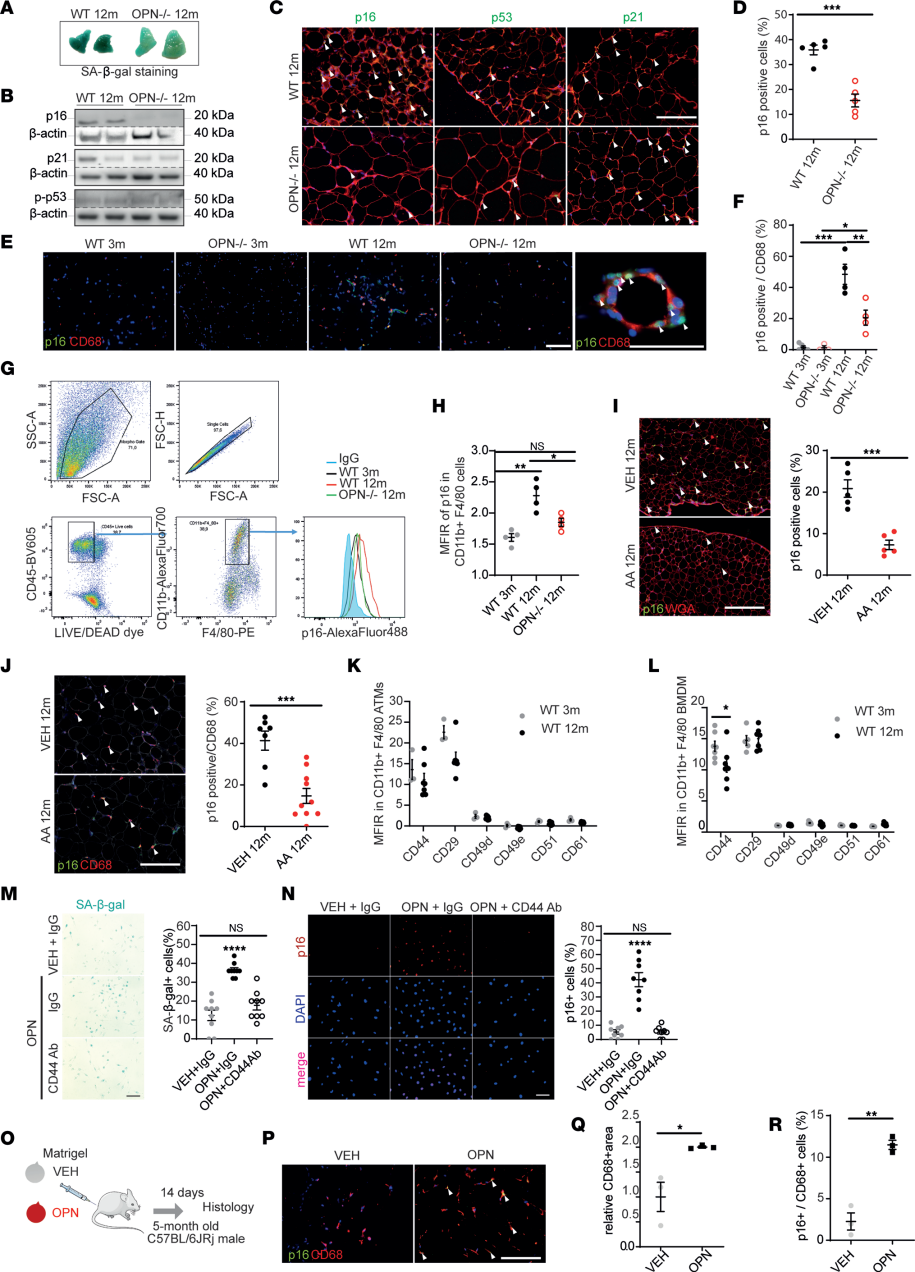
Osteopontin is a critical regulator of adipose tissue and macrophage senescence. (**A**) Representative SA-β-Gal staining of VAT from 12-month-old WT and OPN^–/–^ mice (selected from *n* = 5/group). (**B**) Immunoblot of VAT from 12-month-old WT and OPN^–/–^ mice. Separate loading controls (β-actin) are presented due to the noncontemporaneous run of the same lysates. (**C**) Representative immunofluorescence of VAT (arrowheads: p16, p53, and p21). (**D**) Percentage of p16^+^ cells (*n* = 5/group, same data from the aged WT as in Figure 1). Representative immunofluorescence (**E**) and quantification (**F**) of p16^+^ in the percentage of CD68^+^ cells in VAT (*n* = 4/group). FACS gating strategy (**G**) and quantification (**H**) of p16^+^ in CD11b^+^ F4/80^+^ ATMs from VAT in indicated groups (*n* = 4/group). (**I**) Representative images and quantification (*n* = 5 mice/group) of p16 in VAT of 12-month-old WT treated with AA vs. vehicle (VEH); arrowheads: p16^+^ cells. (**J**) Representative images and quantification of p16^+^ in the percentage of CD68^+^ in VAT (*n* = 7–10 mice/group). Arrowheads: double-positive ATMs. (**K**) Mean fluorescent intensity ratio (MFIR) of OPN receptor expression in CD11b^+^ F4/80^+^ ATMs from VAT of 3- and 12-month-old WT mice (*n* = 3–7 mice/group). (**L**) OPN receptor expression in CD11b^+^ F4/80^+^ ATMs from BMDMs of 3- and 12-month-old WT mice (*n* = 6–7 mice/group). SA-β-Gal (**M**) and p16 (**N**) staining in BMDMs (from 3-month-old WT mice) treated with recombined OPN protein or VEH ± CD44 blocking antibody (*n* = 8–9/condition). (**O**) Protocol of in vivo implantation of Matrigel enriched with OPN protein or VEH. (**P**) Representative immunofluorescence of excised Matrigel after 14 days of implantation (selected from *n* = 3 mice/group). Quantification of relative CD68^+^ area (**Q**) and percentage of p16^+^ cells (**R**) (*n* = 3 mice/group). All scale bars: 50 μm. Data are presented as original images (**A–C**, **E**, **G**, **I**, **J**, **M**, **N**, and **P**) or individual values with mean ± SEM and analyzed with 2-tailed, unpaired Student’s *t* test (**D**, **F**, **I–L**, **Q**, and **R**) or 1-way ANOVA with Tukey’s post hoc test (**F**, **H**, **M**, and **N**); ns, nonsignificant; *P < 0.05, **P < 0.01, ***P < 0.001, ****P < 0.0001.

Accumulation of OPN and other established senescence biomarkers in VAT in an age-dependent manner prompted us to explore a functional role of OPN in VAT senescence by using OPN knockout (OPN^–/–^) mice and pharmacological OPN inhibition by agelastatin A (AA). The experiments comparing young (3-month-old) and aged (12-month-old) WT and transgenic animals were performed contemporaneously. This analysis revealed 4 key points. First, in the VAT of 12-month-old WT mice we found elevated levels of malondialdehyde (a marker of oxidative stress, measured as thiobarbituric acid reactive species), which were prevented by OPN deficiency (Supplemental Figure 2A). Second, compared with that of WT littermates, VAT of 12-month-old OPN^–/–^ mice showed lower SA-β-Gal activity and p16 protein levels (but neither p21 nor pp53; Figure 2, A–D, and Supplemental Figure 2, B and C; we used the same data for p16 from the aged WT as in Figure 1). Third, in 12-month-old WT mice the p16 signal was present in ATMs but blunted by OPN deficiency (Figure 2, E and F), a finding confirmed by FACS analysis (Figure 2, G and H). A similar pattern was observed in CD3^+^ T lymphocytes (Supplemental Figure 2, D and E). Notably, in VAT of 12-month-old OPN^–/–^ mice CD11b^+^ F4/80^+^ macrophages, CD3^+^ and CD4^+^ T cells and NK cells were more populous than in WT littermates (Supplemental Figure 2F) per milligram wet weight. Finally, OPN deficiency protected against age-dependent VAT fibrosis (Supplemental Figure 2G) and glucose intolerance and tended to reduce insulin resistance (Supplemental Figure 2, H and I), despite higher body weight, fat mass, and adipocyte size in OPN^–/–^ mice compared with their WT littermates (Supplemental Figure 2, J–L).

In support of these findings, pharmacological OPN inhibition by AA (19) dramatically reduced p16 expression in VAT and ATMs (Figure 2, I and J) in 12-month-old WT mice. Furthermore, AA treatment improved VAT structure, adipokine secretion profile, and glucose homeostasis (Supplemental Figure 2, M–P).

To confirm a causal role for OPN in VAT senescence, we used exogenous OPN treatment in vitro and in vivo. First, we studied expression levels of OPN receptors in both ATMs and bone marrow–derived macrophages (BMDMs). Because ATMs and BMDMs showed a comparable receptor profile, with predominant expression of CD44 and CD29 (Figure 2, K and L), we used BMDMs from 3-month-old WT mice as surrogate of ATMs to dissect the underlying mechanisms leading to a senescence-like phenotype. Thus, we exposed BMDMs to OPN (Supplemental Figure 2Q). Interestingly, OPN induced SA-β-Gal activity and p16 expression, which could be prevented by CD44 blocking antibody (Figure 2, M and N, and Supplemental Figure 2, Q–T). However, OPN did not induce DNA damage markers γH2AX, p53, or p21 (Supplemental Figure 2U). OPN treatment also induced the monocyte chemoattractant protein *Ccl2* while suppressing *Il1b* expression (Supplemental Figure 2V). OPN treatment induced p16 in OPN^–/–^ BMDMs (Supplemental Figure 2W), further corroborating a receptor-mediated effect. Finally, we confirmed the ability of OPN to (a) increase macrophage recruitment and (b) induce p16 expression in recruited macrophages in young WT mice in vivo with implantation of OPN-enriched (vs. vehicle) Matrigel (Corning Life Sciences; Figure 2, O–R).

Collectively, these results provide evidence for a role of OPN in inducing age-dependent VAT senescence, recruitment of macrophages, and increased p16 expression in ATMs, potentially contributing to impaired VAT structure and function.

### ATMs promote age-dependent VAT and metabolic abnormalities.

**Figure 3:**
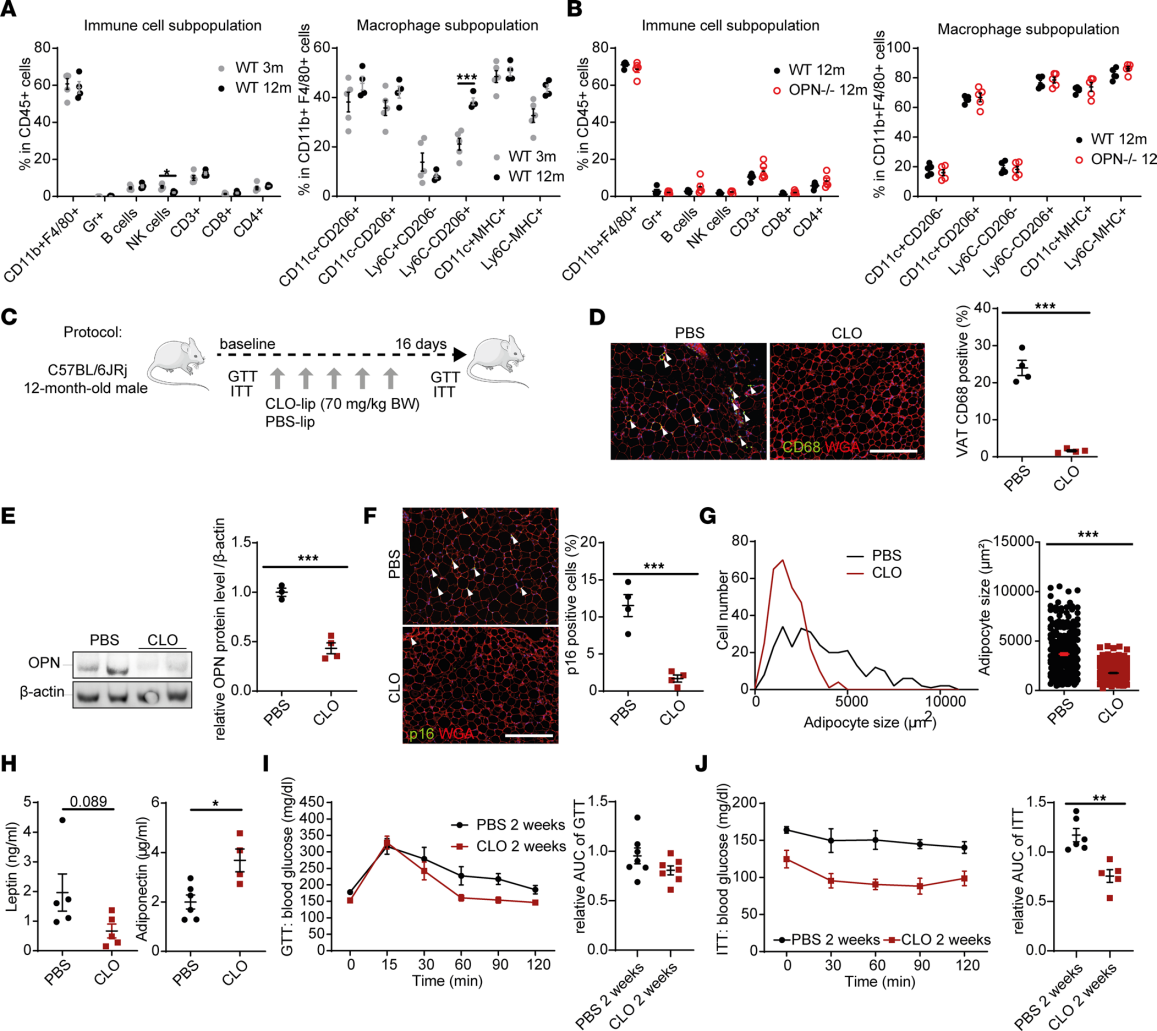
Adipose tissue macrophages promote age-dependent VAT and metabolic abnormalities. (**A**) Immune cell and monocyte/macrophage subpopulations in VAT of 3- and 12-month-old WT mice by FACS (*n* = 3–5/group). (**B**) Immune cell and macrophage subpopulations in VAT of 12-month-old WT and OPN^–/–^ mice by FACS (*n* = 4–5/group). (**C**) Schematic protocol of liposome-clodronate (CLO-lip) treatment. (**D**) Representative CD68 immunofluorescence of VAT of 12-month-old WT mice treated with PBS or CLO (CD68, green; WGA, red; DAPI, blue), and quantification of positive cells (green) in the percentage of total cell number (DAPI; *n* = 4 mice/group). Arrowheads indicate CD68^+^ cells; scale bar: 50 μm. (**E**) Representative OPN Western blot analysis (with β-actin as loading control) and densitometric quantification (*n* = 3–4 mice/group) using protein lysates from VAT in 12-month-old WT mice treated with PBS or CLO. (**F**) Representative p16 immunofluorescence of VAT from 12-month-old WT mice treated with PBS or CLO (p16, green; WGA, red; DAPI, blue) and quantification of p16^+^ cells (green; *n* = 4 mice/group) in the percentage of total cells (DAPI); arrowheads indicate p16^+^ cells, scale bar = 50 μm. (**G**) Distribution and difference of adipocyte size in VAT derived from PBS- or CLO-treated 12-month-old WT mice. (**H**) Plasma adipokine levels by ELISA in PBS- or CLO-treated 12-month-old WT mice (*n* = 4–6 mice/group). (**I** and **J**) Glucose tolerance test (GTT) (**I**) and insulin tolerance test (ITT) (**J**), temporal plot and area under curve (AUC) analysis (*n* = 5–7 mice/group). Data are presented as original images (**D**–**F**) or individual values with mean ± SEM and analyzed with 2-tailed, unpaired Student’s *t* test (**A**, **B**, and **D**–**J**); ns, nonsignificant; *P < 0.05, **P < 0.01, ***P < 0.001.

Our findings revealed a functional link between OPN and a senescence-like ATM phenotype. To further explore whether an age-dependent increase in OPN levels and the ensuing alterations in ATM phenotype promote VAT remodeling and dysfunction, first we used flow cytometry to compare immune populations and ATMs in VAT of 3- vs. 12-month-old WT mice and 12-month-old WT vs. OPN^–/–^ mice based on conventional surface markers (Supplemental Figure 3A) (27, 28). There was no overall difference in VAT-resident immune cell populations between 3- and 12-month-old WT mice or between 12-month-old WT and OPN^–/–^ mice, except a 2-fold diminution in NK cells in 12- vs. 3-month-old WT mice (Figure 3, A and B, left). We also observed a 2-fold increase in the Ly6c^–^CD206^+^ cell population (possessing an M2-like phenotype) that coexpressed the macrophage markers CD11b^+^F4/80^+^ in 12- compared with 3-month-old WT mice (Figure 3, A and B, right).

To test the hypothesis that senescent-like ATMs may propagate dysfunction to neighboring VAT cells, we eliminated them by using liposome-packaged clodronate (CLO) in 12-month-old WT mice (Figure 3C). Having confirmed clearance of ATMs by CLO (Figure 3D and Supplemental Figure 3B), we found significantly lower OPN (Figure 3E) and p16 levels in VAT (Figure 3F). These changes were associated with a sizable improvement in VAT remodeling (decreased adipocyte size), adipokine secretion profile, and glucose tolerance or insulin resistance (Figure 3, G–J).

Collectively, these data reinforce the key role of OPN in inducing a senescence-like ATM phenotype, ultimately promoting age-dependent VAT senescence and metabolic abnormalities.

### OPN-dependent impact of aging on ATM function.

**Figure 4:**
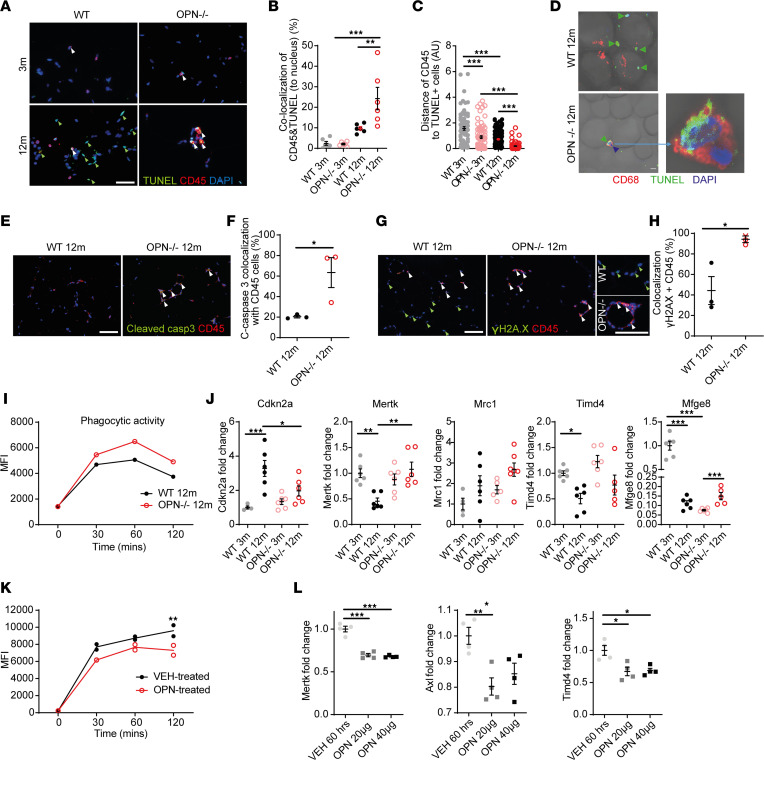
Role of OPN in age-related ATM dysfunction. (**A**) Representative TUNEL/CD45 immunofluorescence of VAT from 3- and 12-month WT and OPN^–/–^ mice. Green arrowheads, single TUNEL^+^ cells; white arrowheads, TUNEL/CD45 double-positive cells. (**B**) Quantification of colocalization of TUNEL^+^ and CD45^+^ cells in percentage of total cell number (*n* = 6 mice/group). (**C**) Quantification of the distance between TUNEL^+^ and CD45^+^ cells (arbitrary units [AU]). (**D**) Representative confocal images of TUNEL/CD68 labeling in fresh VAT of 12-month-old WT and OPN^–/–^ mice (selected from *n* = 3 mice/group). (**E**) Representative cleaved caspase-3/CD45 immunofluorescence of VAT from 12-month WT and OPN^–/–^ mice. Arrowheads, double-positive cells. (**F**) Quantification of colocalization of cleaved caspase-3 and CD45 in percentage of total CD45^+^ cell number (*n* = 3 mice/group). (**G**) Representative γH2AX/CD45 immunofluorescence of VAT from 12-month-old WT and OPN^–/–^ mice. Green arrowheads, single γH2AX^+^ cells; white arrowheads, double-positive cells. (**H**) Quantification of colocalized γH2AX/CD45 double-positive cells in percentage of total CD45^+^ cell number (*n* = 3 mice/group). (**I**) FACS-based quantification of phagocytosis of pHrodo-labeled human leukemia cells as a function of time in BMDMs derived from 12-month-old WT and OPN^–/–^ mice. Line plots denote chronological quantification of MFI of pHrodo green. (**J**) qRT-PCR analysis of senescence and efferocytosis-related gene expression in VAT of 3- and 12-month-old WT and OPN^–/–^ mice (*n* = 4–7 mice/group). (**K**) FACS-based quantification of phagocytosis as a function of time in WT BMDMs treated with OPN protein vs. vehicle. (**L**) qRT-PCR analysis of efferocytosis-related gene expression in WT BMDMs treated with 2 doses of OPN protein (*n* = 4/condition). All scale bars: 50 μm. Data are presented as original images (**A**, **D**, **E**, and **G**) or individual values with mean ± SEM analyzed with 2-tailed, unpaired Student’s *t* test (**F** and **H**) or 1-way ANOVA with Bonferroni post hoc test (**B**, **C**, **J**, and **L**) or 2-way ANOVA (**I** and **K**); ns, nonsignificant; *P < 0.05, **P < 0.01, ***P < 0.001.

One essential ATM function is to eliminate cell debris and apoptotic cells to maintain healthy tissue integrity through a process known as efferocytosis (29). First, we quantified apoptosis (TUNEL positivity) and DNA damage marker (γH2AX) in 3- and 12-month-old WT and OPN^–/–^ mice. We found an age-dependent increase in TUNEL^+^ VAT cells comparable between genotypes, whereas γH2AX^+^ VAT cells increased significantly only in WT mice (Supplemental Figure 4, A and B). Interestingly, in aged OPN^–/–^ mice increased colocalization of TUNEL^+^ and CD45^+^ cells and decreased distance between CD45^+^ and TUNEL^+^ cells suggest better preserved phagocytosis of VAT apoptotic cells compared with aged WT mice (Figure 4, A–C). We confirmed the preserved phagocytic activity of ATMs in VAT of 12-month-old OPN^–/–^ but not in WT mice, with confocal microscopy showing ATMs with two nuclei, one of them being TUNEL^+^ (presumably the nucleus of the phagocytosed cell; Figure 4D). Finally, we found a comparable pattern with cleaved caspase-3 (another apoptosis marker; Figure 4, E and F) and with γH2AX (Figure 4, G and H), which more often colocalized with CD45^+^ cells in 12-month-old OPN^–/–^ but not WT mice, further suggesting that impaired efferocytosis with age depends on OPN.

To demonstrate more directly that OPN compromises efferocytosis, we performed an in vitro phagocytosis assay, comparing BMDMs from 12-month-old WT and OPN^–/–^ mice (Supplemental Figure 4C). This experiment demonstrated impaired phagocytic activity in WT compared with aged-matched OPN^–/–^ BMDMs (Figure 4I and Supplemental Figure 4, D–F). Consistent with this observation, phagocytosis-related gene expression levels were higher in both OPN^–/–^ VAT (Figure 4J) and BMDMs (Supplemental Figure 4G) than in their WT counterparts. Conversely, OPN treatment reduced BMDM phagocytic activity and related gene expression levels compared with vehicle (Figure 4, K and L).

Collectively, these results show that age-related impairment in efferocytosis of senescent-like macrophages is OPN dependent.

### Transplantation of OPN^–/–^ BMDMs rejuvenates VAT and restores metabolic function during aging.

**Figure 5:**
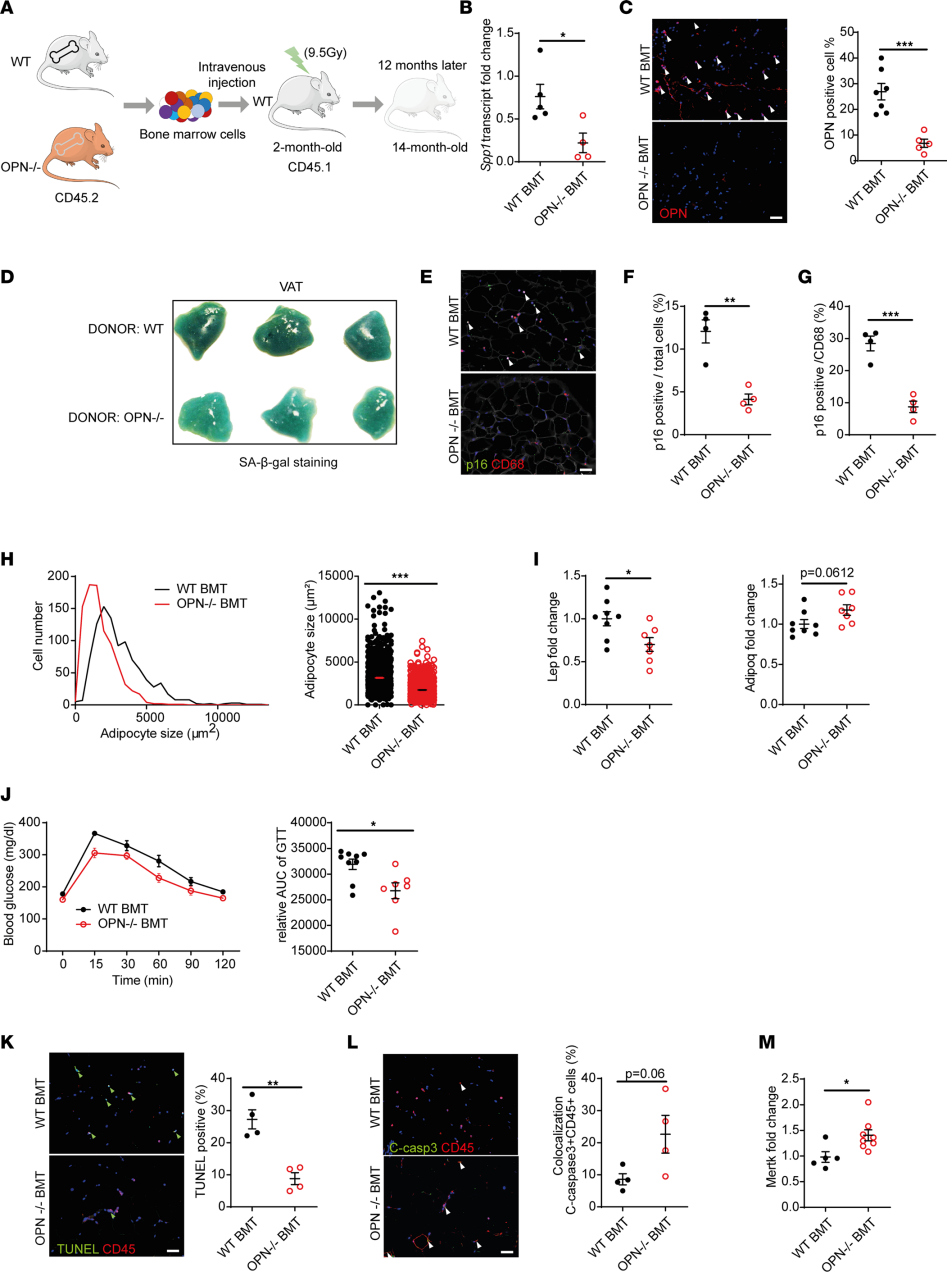
Transplantation of OPN^-/-^ bone marrow rejuvenates VAT and improves metabolic function during aging. (**A**) Protocol of BMT from WT or OPN^–/–^ donor to WT recipient to evaluate the effect of OPN-deficient BMDMs in vivo. (**B**) qRT-PCR analysis of *Spp1* (OPN) in VAT of WT recipients with WT or OPN^–/–^ BMT (*n* = 4–5 mice/group). (**C**) Representative OPN immunofluorescence of VAT of WT recipients with WT or OPN^–/–^ BMT and quantification of positive cells in percentage of total cell number (*n* = 5–7 mice/group). Arrowheads, OPN^+^ cells. (**D**) Representative SA-β-Gal staining in VAT of WT recipients with WT or OPN^–/–^ BMT. (**E**) Representative p16/CD68 immunofluorescence of VAT from WT recipients with WT or OPN^–/–^ BMT. Arrowheads, double-positive cells. (**F** and **G**) Quantification of p16^+^ cells in percentage of total cell number (**F**; *n* = 4 mice/group) or CD68^+^ cell number (**G**; *n* = 4 mice/group). (**H**) Distribution and difference of adipocyte size in VAT from WT or OPN^–/–^ BMT mice. (**I**) qRT-PCR analysis of adipokine gene expression (leptin, *Lep*; adiponectin, *Adipoq*) in VAT of WT or OPN^–/–^ BMT mice (*n* = 7–8 mice/group). (**J**) Glucose tolerance test, temporal plot, and area under curve (AUC) analysis (*n* = 7–9 mice/group). (**K**) Representative TUNEL/CD45 immunofluorescence of VAT from WT or OPN^–/–^ BMT mice and quantification of TUNEL^+^ cells (green arrowheads) in percentage of total cell number (*n* = 4 mice/group). (**L**) Representative cleaved caspase-3/CD45 immunofluorescence and quantification in VAT of the same animals as above. Arrowheads, double-positive cells (*n* = 4 mice/group). (**M**) qRT-PCR analysis of *Mertk* in VAT of WT and OPN^–/–^ BMT mice (*n* = 5–8 mice/group). All scale bars: 50 μm. Data are presented as original images (**C**–**E**, **K**, and **L**) or individual values with mean ± SEM and analyzed with 2-tailed, unpaired Student’s *t* test (**B**, **C**, and **F**–**M**); ns, nonsignificant; *P < 0.05, **P < 0.01, ***P < 0.001.

Resident ATMs appear to be replaced with BMDMs over time (30). To explore whether there is a significant age-dependent myeloid cell–derived (vs. local self-renewal) contribution to ATMs that through altered properties may instigate VAT senescence and dysfunction, we conducted bone marrow transplantation (BMT). After total body irradiation, WT recipients (CD45.1) underwent BMT with donors of 2-month-old WT and OPN^–/–^ mice (CD45.2) and were subsequently monitored until the age of 14 months (Figure 5A). To confirm efficient BMT, we observed that blood leukocytes and CD45^+^ cells in VAT were reconstituted with donor-derived cells (CD45.2^+^) over 95%, without affecting their number (Supplemental Figure 5, A–C). OPN gene expression and OPN^+^ cells in VAT markedly decreased after OPN^–/–^ BMT (Figure 5, B and C), suggesting that myeloid cell–derived leukocyte (mostly macrophage) recruitment is a primary source of OPN in the aged VAT. Notably, after OPN^–/–^ BMT, VAT SA-β-Gal staining slightly decreased compared with WT BMT (Figure 5D), and so did the number of p16^+^ cells and specifically p16^+^ ATMs (Figure 5, E–G) but not p16^+^ CD3^+^ cells (Supplemental Figure 5D). Blunted expression of senescence biomarkers and OPN in OPN^–/–^ BMT VAT was associated with smaller adipocyte size, normalized adipokine gene expression pattern, and increased glucose tolerance (Figure 5, H–J). Finally, OPN^–/–^ BMT reduced TUNEL^+^ cells (Figure 5K) and increased colocalization of cleaved caspase-3^+^ CD45^+^ cells in VAT (Figure 5L), along with higher efferocytosis-related gene expression (Figure 5M).

Collectively, our results indicate that an age-dependent myeloid cell-derived OPN exerts an important role in promoting VAT senescence and dysfunction.

## Discussion

In this study, we showed that age-related VAT remodeling and senescence are strongly related to a senescence-like ATM phenotype characterized by an impairment of major macrophage functions, such as efferocytosis. Importantly, we uncovered a key role for the matricellular protein OPN to drive these processes.

### VAT senescence during aging.

Our data indicated that VAT remodeling occurs during the aging process together with cellular senescence. Indeed, time course analysis of WT mice between 2 and 24 months of age indicated that VAT senescence starts at the age of 12 months, earlier than previously reported (25, 26). VAT aging is associated with structural remodeling (i.e., enlargement of adipocytes, fibrotic deposition), metabolic dysfunction (i.e., impaired glucose tolerance, insulin sensitivity, and adipokine secretion), and senescence. To assess VAT cellular senescence, we found increased expression of several biomarkers, including SA-β-Gal activity, DNA damage response (γH2A.X, H3K9me3), expression of p53 and cyclin-dependent kinase inhibitors (p21, p16), and impaired proliferation (EdU incorporation). This senescence pattern was specific to VAT, as shown by the different profile of senescence markers induced in SAT, where p21 and H3K9me3 were upregulated with age, whereas p16 was not. Lack of p16 induction in the aged SAT coincided with the absence of OPN expression. From these observations, we hypothesized a causal relationship between OPN and p16 induction, occurring as a site-specific event that we investigated further in detail. On the other hand, we attributed the comparable age-related induction of p21 and H3K9me3 in both VAT and SAT to systemic effects of aging, such as increased phenylalanine levels, as we have recently reported for the aged heart (31). Although in the present study we explored OPN-related effects occurring in VAT, we cannot exclude clinically relevant consequences of SAT senescence, as have been recently suggested for both mice and humans by our group and others (7, 32, 33).

Interestingly, we identified both senescence marker and matricellular protein (*Spp1*, *Thbs1*) transcripts as being more discriminative than proinflammatory genes to drive VAT aging. In parallel, efferocytosis-related genes were strongly downregulated. In line with the differential age-dependent expression pattern of OPN and p16, we identified p16 as the unique senescence marker being induced by OPN, a member of the matricellular protein family (20). Thus, we focused on p16, a reliable senescence biomarker, which recently showed the strongest association with aging (1–30 months) in the Mouse Aging Cell Atlas (34). In support of the role of p16, targeted removal of p16-expressing cells in aged mice dramatically reduces senescent cell burden, with marked improvement in health and extended life span (1, 35).

Another finding is that genetic OPN deletion resulted in significantly larger adipocyte size and VAT mass despite their better metabolic function. Our results highlight a critical involvement of OPN in inducing VAT senescence, confirming our initial report that VAT was the major source of OPN during aging (19) and extending its recognized role in obesity-related VAT remodeling (7, 22) to aging.

### Importance of macrophage senescence in VAT aging.

Interestingly, in naive macrophages OPN reduced phagocytic activity (27, 36), which is considered one of the key events mediating VAT dysfunction. These in vitro findings were supported by in vivo genetic deletion and pharmacological inhibition of OPN, which reduced VAT senescence while restoring a major macrophage function, i.e., efferocytosis. Furthermore, aging was associated with multiple characteristics of cellular senescence, such as reduced replication, increased DNA damage, increased SA-β-Gal activity, and upregulation of p16 and p21 in ATMs. We may speculate that such features of senescence were induced by the changing VAT microenvironment, through SASP along with increased levels of ROS in aged WT but not OPN^–/–^ VAT.

To dissect the interplay between OPN and senescence-like features in ATMs, we explored how OPN may spread senescence to the adjacent tissue, ultimately leading to VAT remodeling and dysfunction during the aging process (6, 13, 16). Indeed, ATMs, the most abundant AT immune cell type in natural aging, are particularly important given their key role in AT homeostasis and functional maintenance (9). By contrast, ATMs may have harmful effects under stress conditions, such as overnutrition, responsible for a chronic, low-grade proinflammatory state (37). We established that aging induced p16 upregulation in ATMs of WT mice, which was blunted by OPN deficiency and CLO liposome–mediated clearance of ATMs, together with AA, a compound with OPN inhibitory properties (38). AA can exert pleiotropic effects through its effective blocking of protein synthesis (39); therefore, we cannot safely exclude extraneous effects on effector molecules other than OPN. This caveat notwithstanding, all these interventions ameliorated VAT structural remodeling (i.e., adipocyte hypertrophy) and metabolic function (i.e., systemic glucose tolerance and adipokine secretion profile).

Next, we demonstrated an impairment of ATM efferocytosis regulated by OPN both in vivo and in vitro, putatively mediated by CD44 OPN receptor. Indeed, efferocytosis, which is crucial in maintaining AT homeostasis by removing apoptotic cells and cell debris, was impaired, as observed by lower engulfment of TUNEL^+^ or cleaved caspase-3^+^ cells in aged WT but not in OPN^–/–^ mice. In vitro functional assay further indicated that aged OPN^–/–^ BMDMs exerted preserved phagocytic activity with higher expression levels of key efferocytosis-related genes, such as *Mertk* (40, 41). On the other hand, BMT derived from OPN^–/–^ mice (vs. WT BMT) was efficient to blunt the expression of senescence biomarkers in aged VAT and restore adipocyte size and adipokine expression. From these observations we may infer the importance of recruited, BMDMs in the control of aged VAT microenvironment.

Most recent publications mentioned favorable effects by systemic elimination of senescent cells with senolytic drugs (1, 26, 35, 42). On the other hand, induction of senescence in some cell types is beneficial during aging and repair processes (19, 43–45). Beside senolytic drugs, we have identified OPN as a novel target for ameliorating tissue homeostasis, complementing our previous observations made in the aging heart (19) and fatty liver (46). Collectively, our results showed that elimination of senescent-like ATMs improved AT remodeling and dysfunction, suggesting that macrophage-targeted therapeutics (e.g., macrophage-directed pharmacological OPN inhibition) could be a viable strategy to control VAT senescence and subsequent systemic metabolic alterations resulting from aging.

## Methods

### Mice.

C57BL/6JRj mice (referred to as WT) were purchased from Janvier Labs. OPN-knockout mice (B6.129S6(Cg)-Spp1^tm1Blh^/J), purchased from The Jackson Laboratory, had been backcrossed with C57/BL6J mice more than 10 generations and were bred by heterozygous mice to yield homozygous (referred to as OPN^–/–^) and WT littermates. The p16-luciferase mice (strain code 01XBT--B6.Cg-Cdkn2a tm3.1Nesh Tyr c-2J/Nci) were obtained from the National Cancer Institute mouse repository (courtesy of Norman Sharpless, University of North Carolina, Chapel Hill, North Carolina, USA) (25). All mice were housed in a pathogen-free platform at a constant temperature (22°C), with a 12-hour light-dark cycle and unrestricted access to a chow diet (CD, A0310; Safe Diets) and water. Chow diet composition was 13.5% fat, 61.3% carbohydrate, and 25.2% protein. During follow-up, animals underwent monthly body weight evaluation. The experiments comparing the young (3-month-old) and aged (12-month-old) WT and transgenic animals were performed contemporaneously.

### In vivo luciferase detection.

Luciferase activity was detected and analyzed in collaboration with Johanne Seguin, Paris Descartes University, as previously described (25). Briefly, mice were anesthetized with isoflurane and then injected intraperitoneally with D-luciferin substrate (Caliper Life Sciences; 15 mg/mL in PBS). Images were taken with the PhotonIMAGER system (Biospace Lab). After substrate administration, peak luminescence was measured at 6–8 minutes. For simultaneous image capture of more than 3 animals, a wide-angle lens was used after injection of 10 μL/g body weight of substrate. To compare multiple images taken at the same exposure point, PhotoAcquisition Software for PhotonIMAGER was used after D-luciferin administration, and acquisition lasted for 10 minutes (from *t* = 10 min to *t* = 20 min after luciferin injection). An additional region of interest was used to normalize for background luminescence on each image for all analyses.

### Glucose tolerance test.

Mice were fasted for 6–7 hours and then given glucose solution intraperitoneally (1.5 mg/g body weight). Blood samples were collected from tail vein at baseline and 15, 30, 60, 90, and 120 minutes after injection, and glucose concentration was measured with an automatic glucometer (Accu-Chek Performa, Roche).

### Insulin tolerance test.

Mice were fasted for 6–7 hours and then given insulin solution intraperitoneally (0.5 mU/g of body weight; Humulin, Lilly). Blood samples were collected from tail vein at baseline and 30, 60, 90, and 120 minutes after injection, and glucose concentration was measured as above.

### In vivo matrigel plug assay.

OPN-enriched in vivo Matrigel plug assay was performed according to the manufacturer’s instructions (Matrigel Basement Membrane Matrix, catalog 354234, Corning) and previous report (47). A total of 6 male 5-month-old WT mice were used. In brief, 500 μL Matrigel (catalog 354234, Corning) was mixed with 100 μL of 50 μg mouse recombinant OPN (R&D Systems) or PBS (as vehicle). Mixed solution was subcutaneously injected to the groin of anesthetized mice. After 14 days, the Matrigel plug was removed, fixed in 4% formaldehyde with mesh cassettes (Trajan), and further processed for immunohistochemistry.

### In vivo macrophage depletion.

A total of 17 12-month-old male WT mice weighing 30–36 g were used. Before in vivo macrophage depletion, mice underwent baseline assessment of metabolic function (glucose and insulin tolerance tests). Mice were then injected intraperitoneally with 70 mg/kg CLO in liposomes or PBS control liposomes (ClodLip BV) every 4 days for 4 weeks (70% dose used in reference) (48). Body weights were recorded at the same time. After final metabolic assessments, mice were euthanized by cervical dislocation, with organs and bloods harvested and processed for further evaluation.

### OPN inhibition by small molecule.

A total of 20 11-month-old male WT mice, weighing 28–35 g, were used. AA, a small organic molecule known to inhibit OPN production in vivo, was synthesized and provided by Takehiko Yoshimitsu (Graduate School of Pharmaceutical Sciences, Okayama University). After baseline assessment of metabolic function (glucose and insulin tolerance tests), mice were given 1.5 mg/kg of AA or vehicle only (2-hydroxypropyl-β-cyclodextrin solution; Sigma-Aldrich) intraperitoneally every 4 days for 4 weeks (19). After serial recording of body weight and final metabolic assessments, mice were euthanized by cervical dislocation, followed by harvesting and processing of organs and blood.

### Mouse BMT.

Whole-body irradiated (9.5 Gy) 8-week-old male WT mice (CD45.1: B6.SJL-Ptprca Pepcb/BoyCrl, congenic strains, C57BL/6 background) were purchased from Charles River Laboratories as recipient. Bone marrow cells were obtained from femurs of donor mice (4-month-old male WT and OPN^–/–^ mice as described above; CD45.2). One day after irradiation, recipient mice received 2 million donor bone marrow cells by intravenous tail injection. Mice were kept and aged in house until reaching 14 months of age. Body weight was recorded monthly, and peripheral blood reconstitution was analyzed by FACS at 2, 4, 5, 9, and 12 months after BMT. Antibodies and fluorophores used are listed in the Supplemental Table.

### Plasma adipokine measurement.

Whole blood samples were centrifuged (5,000*g*, 10 min at 4°C), and plasma fraction was stored at –80°C. Plasma levels of adipokines were assessed with a Magnetic Luminex screening assay kit (adiponectin, LXSAMSM-1; leptin, LXSAMSM-19; Bio-Techne) according to the manufacturer’s instructions.

### Tissue processing and histology.

Organs were fixed with 4% formaldehyde solution (Sigma-Aldrich) immediately and subjected to paraffin embedding after a minimum of 1 week’s fixation. After deparaffinization, 5 μm–thick AT sections were stained with hematoxylin and eosin (Sigma-Aldrich) for general morphology and adipocyte size measurement, and Sirius Red for tissue fibrosis measurement, respectively.

For immunofluorescence, rehydrated sections were subjected to heat-mediated antigen retrieval (citrate buffer pH 6.0, Dako Products) and blocking with 30% goat serum. Sections were incubated with primary antibodies diluted in antibody diluent (Dako Products) overnight at 4°C in a humidified chamber. Secondary antibodies conjugated with fluorescent dyes were used in combination with DAPI for visualization. The AT staining for p16 used the same data from the aged WT cohort in Figures 1 and 2.

Pictures stained for hematoxylin and eosin and Sirius Red were captured with an Axioplan 2 Imaging microscope (Zeiss). Fluorescent images were acquired with an Axioplan M2 Imaging microscope (Zeiss). For adipocyte size, at least 200 cells per sample were traced and quantified surface area. For interstitial fibrosis, 3–5 images per sample were measured and normalized to total surface area with ImageJ software (NIH). For quantification, images obtained from the same animal were averaged and plotted.

### SA-β-Gal staining.

VAT samples cut into 5 mm × 5 mm pieces or cultured macrophages were first fixed in 2% formaldehyde and 0.2% glutaraldehyde, then subjected to staining solution (1 mg/mL X-gal, Thermo Fisher Scientific; and 5 mM potassium ferrocyanide, 5 mM potassium ferricyanide, 40 mM citric acid, 40 mM phosphate sodium, 150 mM NaCl, and 2 mM MgCl_2_, all from Sigma-Aldrich), and kept at 37°C for 12 hours. The images were taken with a GR digital camera (Ricoh).

### TUNEL staining.

TUNEL staining was performed according to the manufacturer’s instructions (ApopTag Plus In Situ Apoptosis Fluorescein Detection Kit, MilliporeSigma) with minor modifications. Briefly, deparaffinized sections were subjected to proteinase K treatment (20 mg/mL; Qiagen), and incubated with TdT enzyme for 1 hour. After the reaction, was stopped, digoxigenin-conjugated fluorescein was applied to visualize the signals. CD45 immunohistochemistry and DAPI counterstaining were performed to show the TUNEL^+^ cell localization.

### Quantitative PCR.

Total RNA was extracted from 80 mg of powdered AT samples with an RNeasy Fibrous Tissue Kit (Qiagen). RNA from cultured macrophages was extracted with an RNeasy Mini Kit (Qiagen). First-strand DNA was synthesized from 0.5–1.0 μg total RNA with a High-Capacity cDNA Reverse Transcription Kit (Applied Biosystems) and a SimpliAmp thermal cycler (Applied Biosystems) according to the manufacturer’s instructions. Quantitative reverse transcription PCR (qRT-PCR) was performed and analyzed with TaqMan Universal PCR Master Mix and StepOne Real-Time PCR System (Applied Biosystems). β-Actin was used as internal control. Taqman oligos are listed in the Supplemental Table.

### Western blot analysis.

We homogenized 80–90 mg of AT in “Platonic” urea lysis buffer containing 7 M urea, 10% glycerol (v/v), 10 mM Tris-HCl (pH 6.8), 1% SDS, and 1 mM dithiothreitol, supplemented with protease and phosphatase inhibitor cocktail tablets (Pierce/Thermo Fisher Scientific). After vortexing, lysates were sonicated, passed through a 21-gauge needle at least 6 times, rotated for 30 minutes at 4°C, and spun to obtain supernatant. Protein concentration was adjusted according to the Bradford method (Bio-Rad). Denatured total protein (20 μg) was loaded onto 10% SDS-PAGE. Separated proteins were transferred to PVDF membranes (Invitrogen), blocked in Pierce Clear Milk Blocking Buffer (Thermo Fisher Scientific) and probed for selected proteins with a specific primary antibody at 4°C overnight, followed by incubation with the corresponding HRP-conjugated secondary antibodies (Abcam). In some figures separate loading controls (β-actin) are presented because of the noncontemporaneous run of the same lysates. Densitometric quantification was performed with ImageJ software, and expression was normalized to β-actin levels. Primary antibodies are listed in the Supplemental Table.

### AT immune cell and macrophage flow cytometric analysis (FACS).

AT was kept on ice and minced to 1–3 mm pieces in heparinized ice-cold PBS. After snap vortexing, PBS was removed, and tissues were digested with collagenase II solution at a concentration of 1 mg/mL (Sigma-Aldrich) in a 37°C water bath with gentle shaking for 20 minutes. Digested tissue was then passed through 70 μm mesh and centrifuged to recover stromal vascular fraction (SVF). After RBC lysis (ACK Lysing Buffer, Gibco), SVF cells were suspended in PBS supplemented with 2% FCS and stained with appropriate antibodies for surface marker and isotype controls for 30 minutes at 4°C, avoiding light. Antibodies and fluorophores are listed in the Supplemental Table. To gate out dead cells, we used LIVE/DEAD Fixable Dead Cell Stain Kits (Invitrogen) to label SVF derived from VAT of 3- and 12-month-old WT mice as well as 12-month-old OPN^–/–^ mice. We specifically selected living hematopoietic cells (CD45^+^ live cells) to identify the macrophage subpopulation (CD11b^+^F4/80^+^). For intracellular p16 staining, SVF cells stained with surface markers were fixed and permeabilized with Cytofix/Cytoperm solution (BD Biosciences). Cells were stained with p16 antibody (Abcam) or corresponding isotype control followed by incubation with biotinylated goat anti-mouse antibody and streptavidin Alexa Fluor 488, using a Mouse on Mouse detection kit (Vector Laboratories) as instructed by the manufacturer. Flow cytometry was performed with a LSR II (BD Biosciences). Data were evaluated and analyzed with FlowJo. The mean fluorescent intensity ratio of p16 was calculated via the equation mean fluorescent intensity (MFI) of p16/MFI of IgG.

### Proliferation assay.

We injected 10 μg/g body weight of EdU (Life Technologies) intraperitoneally into 3- or 12-month-old WT and OPN^–/–^ mice. One 3-month-old WT mouse was injected with PBS to act as a background control. Twenty-four hours later mice were euthanized. VAT was digested and the SVF was isolated. SVF cells stained with surface markers were fixed, and the Click-iT Plus EdU flow cytometry assay kit (Life Technologies) was used to detect EdU^+^ cells. Flow cytometry and analysis were performed as described above.

### Generation of BMDMs.

Bone marrow cells were isolated from femurs and tibias of 3- or 12-month-old WT and OPN^–/–^ mice. After RBC lysis (LCK buffer, Sigma-Aldrich) and passage through a 70 μm mesh, bone marrow cells were plated with RPMI 1640 medium (Gibco) supplemented with 5% FCS, 1% penicillin/streptomycin (Gibco), and 30 ng/mL macrophage colony-stimulating factor (R&D Systems). Five days after differentiation into macrophages, the cells were treated with Accutase (Sigma-Aldrich), counted, and replated at 1.5 × 10^5^/well in 12-well noncoated plate. The BMDMs were further subjected to OPN stimulation experiment or phagocytosis assay.

### BMDM stimulation with OPN.

Modulation of BMDM function with OPN protein was evaluated as previously described with minor modifications (49). Briefly, we prepared OPN solution 20 or 40 μg of mouse recombinant OPN (R&D Systems) dissolved in 5 mL of 1× PBS. Then 400 μL of either of these solutions was used to coat wells of 12-well plates (designated as 20 or 40 μg OPN), and 75 μL of either OPN solution was used to coat chamber slides for 4 hours at room temperature and subsequently stabilized with 0.5% polyvinylpyrrolidone (Sigma-Aldrich) for 1 hour at room temperature. BMDMs (1.5 × 10^5^/well) were seeded onto plates in RPMI 1640 medium supplemented with 5% FCS and 30 ng/mL macrophage colony-stimulating factor. For antibody inhibition, BMDMs were first incubated with 10 μg/mL of rat monoclonal anti-CD44 antibody (Novus Biologicals, catalog NBP2-22530) or isotype control at room temperature for half an hour before seeding onto an OPN-coated plate. Twenty-four or 36 hours later, cells were harvested for RNA extraction, immunofluorescence, or SA-β-Gal staining.

### BMDM immunofluorescent staining.

BMDMs were passed onto an OPN-coated 8-well Lab-Tek II chambered cover glass (Nalge Nunc International) at 1.5 × 10^4^/well density. After incubation as described above, BMDMs were washed with chilled PBS buffer and fixed with 2% paraformaldehyde solution for 10 minutes at room temperature and permeabilized with 0.05% Triton-X (Sigma-Aldrich). Cells were blocked with 2% BSA solution (Sigma-Aldrich) and subjected to p16 and F4/80 antibody treatment overnight. Corresponding secondary antibodies conjugated with fluorescent dyes were used to visualize signal with DAPI counterstaining. Images were acquired as described above.

### Phagocytosis assay.

Cells from the human leukemia cell line, HL60 (ATCC), were labeled with pHrodo iFL Green STP Ester (amine reactive) (Thermo Fisher) as previously described (40), with minor modification. Briefly, HL60 cells were washed twice with PBS buffer and incubated at room temperature for 30 minutes with 0.1 μg/mL pHrodo. Cells were washed with PBS buffer twice and resuspended in RPMI medium containing 0.5% FCS at a density of 4.0 × 10^5^/mL to be used for engulfment. To 5.0 × 10^4^ BMDMs in 500 μL of RPMI medium containing 5% FCS in a 12-well plate, 2.0 × 10^5^ pHrodo-labeled HL60 cells were added and incubated at 37°C for 0, 30, 60, and 120 minutes. Cells were detached from the plate by trypsin and 1 mM EDTA and collected by centrifugation. Cells were labeled with surface markers CD11b and F4/80, then resuspended in 500 μL of PBS buffer containing 2% FCS and analyzed by flow cytometry with LSR II (BD Biosciences). Data were evaluated and analyzed with FlowJo. For time-lapse imaging, 1.5 × 10^4^/well BMDMs were passed onto an 8-well Lab-Tek II Chambered Coverglass (Nalge Nunc) and incubated at 37°C for 0, 30, 60, and 120 minutes with 6 × 10^4^ pHrodo-labeled HL60 cells in 250 μL of RPMI medium containing 5% FCS and observed by fluorescence microscopy.

### Discriminative gene analysis.

In addition to exploring individual changes involving various molecular targets, we aimed to also evaluate the contextual importance of the changes affecting major themes that characterize the AT functional signature under the effect of aging. To this purpose we measured the VAT expression profile of a panel of 43 genes selected to illustrate 6 major themes characterizing the functional signature of the AT: efferocytosis, matricellular proteins, senescence, inflammation, AT function, and secretome. A random forest supervised learning algorithm was applied iteratively to evaluate and rank the relative discriminative value (i.e., feature importance) associated with changes in these gene expression profiles induced by aging. These computations were performed using the R environment for statistical computing and the algorithmic implementation available in the “randomForest” R package (version 4.6–12). To assess the relative discriminative value associated with each measured expression profile, we averaged the feature importance provided by the random forest algorithm over 10,000 successive iterations performed for each of the analyzed situations. The individual discriminative value of each transcriptional profile was expressed as percentages of the total discriminative power of the panel of selected transcriptional descriptors. We plotted the resulting values after grouping them by functional category and ranking the available categories in decreasing order of their strongest transcriptional descriptor, to illustrate the contextual importance of the functional changes induced by VAT aging.

### Statistics.

All data are presented as mean ± SEM. Difference between 2 groups was analyzed by unpaired, 2-tailed *t* test. Comparisons between multiple groups were performed via 1- or 2-way ANOVA followed by Tukey’s or Bonferroni’s test. These analyses were performed in GraphPad Prism 5.0. *P* < 0.05 was considered significant.

### Study approval.

All animal experiments were approved by the Institutional Animal Care and Use Committee of the French National Institute of Health and Medical Research (INSERM) U955 (ComEth 15-001) and Institut Gustave Roussy’s Institutional Animal Care and Use Committee (Villejuif, France).

## Author contributions

DS, YZ, AM, and MP designed, conducted most of the experiments and data analysis, and prepared the manuscript; ZM, L Lipskaia, SN, HL, MH, and TD helped with organ isolation and imaging experiments; L Lamendour and DMA performed gene and protein expression analysis; MB assisted with mouse work and live imaging experiments; MS performed plasma analysis; DR, TY, and PG donated materials and provided helpful suggestions; FL and SA provided helpful suggestions and contributed to the manuscript; CH performed gene discriminative analysis; and GC and GD designed and supervised this work and helped write the manuscript. DS, YZ, AM, and MP equally contributed as first authors. Order assignment among them was decided as follows: DS was assigned first because he designed most of the initial study protocol and was involved in all experiments. YZ was assigned second because her contribution to the macrophage analysis was critical. AM was assigned third because her contribution to the extensive revision work was crucial. MP was assigned fourth because her contribution to the first version of the manuscript was important.

## Supplementary Material

Supplemental data

## Version 1 04/24/2023

Electronic publication

## References

[1] Palmer AK (2019). Targeting senescent cells alleviates obesity-induced metabolic dysfunction. Aging Cell.

[2] Lumeng CN (2008). Phenotypic switching of adipose tissue macrophages with obesity is generated by spatiotemporal differences in macrophage subtypes. Diabetes.

[3] Pini M (2021). Adipose tissue senescence is mediated by increased ATP content after a short-term high-fat diet exposure. Aging Cell.

[4] Rabhi N (2022). Obesity-induced senescent macrophages activate a fibrotic transcriptional program in adipocyte progenitors. Life Sci Alliance.

[5] Matacchione G (2022). Senescent macrophages in the human adipose tissue as a source of inflammaging. Geroscience.

[6] Minamino T (2009). A crucial role for adipose tissue p53 in the regulation of insulin resistance. Nat Med.

[7] Rouault C (2021). Senescence-associated β-galactosidase in subcutaneous adipose tissue associates with altered glycaemic status and truncal fat in severe obesity. Diabetologia.

[8] Briot A (2018). Senescence alters PPARγ (peroxisome proliferator-activated receptor gamma)-dependent fatty acid handling in human adipose tissue microvascular endothelial cells and favors inflammation. Arterioscler Thromb Vasc Biol.

[9] Elder SS, Emmerson E (2020). Senescent cells and macrophages: key players for regeneration?. Open Biol.

[10] Kale A (2020). Role of immune cells in the removal of deleterious senescent cells. Immun Ageing.

[11] Munoz-Espin D, Serrano M (2014). Cellular senescence: from physiology to pathology. Nat Rev Mol Cell Biol.

[12] Sun K (2011). Adipose tissue remodeling and obesity. J Clin Invest.

[13] Tchkonia T (2010). Fat tissue, aging, and cellular senescence. Aging Cell.

[14] Kuk JL (2009). Age-related changes in total and regional fat distribution. Ageing Res Rev.

[15] Trim W (2018). Parallels in immunometabolic adipose tissue dysfunction with ageing and obesity. Front Immunol.

[16] Hall BM (2016). Aging of mice is associated with p16(Ink4a)- and β-galactosidase-positive macrophage accumulation that can be induced in young mice by senescent cells. Aging (Albany NY).

[17] Hall BM (2017). p16(Ink4a) and senescence-associated β-galactosidase can be induced in macrophages as part of a reversible response to physiological stimuli. Aging (Albany NY).

[18] Liu JY (2019). Cells exhibiting strong p16^INK4a^ promoter activation in vivo display features of senescence. Proc Natl Acad Sci U S A.

[19] Sawaki D (2018). Visceral adipose tissue drives cardiac aging through modulation of fibroblast senescence by osteopontin production. Circulation.

[20] Frangogiannis NG (2012). Matricellular proteins in cardiac adaptation and disease. Physiol Rev.

[21] Shirakawa K (2016). Obesity accelerates T cell senescence in murine visceral adipose tissue. J Clin Invest.

[22] Nomiyama T (2007). Osteopontin mediates obesity-induced adipose tissue macrophage infiltration and insulin resistance in mice. J Clin Invest.

[23] Shimatani K (2009). PD-1+ memory phenotype CD4+ T cells expressing C/EBPalpha underlie T cell immunodepression in senescence and leukemia. Proc Natl Acad Sci U S A.

[24] Saker M (2016). Osteopontin, a key mediator expressed by senescent pulmonary vascular cells in pulmonary hypertension. Arterioscler Thromb Vasc Biol.

[25] Burd CE (2013). Monitoring tumorigenesis and senescence in vivo with a p16(INK4a)-luciferase model. Cell.

[26] Baker DJ (2016). Naturally occurring p16(Ink4a)-positive cells shorten healthy lifespan. Nature.

[27] Silva HM (2019). Vasculature-associated fat macrophages readily adapt to inflammatory and metabolic challenges. J Exp Med.

[28] Daemen S, Schilling JD (2019). The interplay between tissue niche and macrophage cellular metabolism in obesity. Front Immunol.

[29] Henson PM (2017). Cell removal: efferocytosis. Annu Rev Cell Dev Biol.

[30] Geissmann F (2003). Blood monocytes consist of two principal subsets with distinct migratory properties. Immunity.

[31] Czibik G (2021). Dysregulated phenylalanine catabolism plays a key role in the trajectory of cardiac aging. Circulation.

[32] Espinosa De Ycaza AE (2021). Senescent cells in human adipose tissue: a cross-sectional study. Obesity (Silver Spring).

[33] Gao Z (2020). Age-associated telomere attrition in adipocyte progenitors predisposes to metabolic disease. Nat Metab.

[34] Tabula Muris Consortium (2020). A single-cell transcriptomic atlas characterizes ageing tissues in the mouse. Nature.

[35] Xu M (2018). Senolytics improve physical function and increase lifespan in old age. Nat Med.

[36] Jaitin DA (2019). Lipid-associated macrophages control metabolic homeostasis in a Trem2-dependent manner. Cell.

[37] McNelis JC, Olefsky JM (2014). Macrophages, immunity, and metabolic disease. Immunity.

[38] Mason CK (2008). Agelastatin A: a novel inhibitor of osteopontin-mediated adhesion, invasion, and colony formation. Mol Cancer Ther.

[39] McClary B (2017). Inhibition of eukaryotic translation by the antitumor natural product agelastatin A. Cell Chem Biol.

[40] Nishi C (2014). Tim4- and MerTK-mediated engulfment of apoptotic cells by mouse resident peritoneal macrophages. Mol Cell Biol.

[41] Zagorska A (2014). Diversification of TAM receptor tyrosine kinase function. Nat Immunol.

[42] Baker DJ (2011). Clearance of p16Ink4a-positive senescent cells delays ageing-associated disorders. Nature.

[43] Xue W (2007). Senescence and tumour clearance is triggered by p53 restoration in murine liver carcinomas. Nature.

[44] Krizhanovsky V (2008). Senescence of activated stellate cells limits liver fibrosis. Cell.

[45] He S, Sharpless NE (2017). Senescence in health and disease. Cell.

[46] Remmerie A (2020). Osteopontin expression identifies a subset of recruited macrophages distinct from Kupffer cells in the fatty liver. Immunity.

[47] Perino A (2014). TGR5 reduces macrophage migration through mTOR-induced C/EBPβ differential translation. J Clin Invest.

[48] Westwell-Roper CY (2014). Resident macrophages mediate islet amyloid polypeptide-induced islet IL-1β production and β-cell dysfunction. Diabetes.

[49] Frolova EG (2010). Thrombospondin-4 regulates vascular inflammation and atherogenesis. Circ Res.

