# Supplementary material for: Osteopontin promotes age-related adipose tissue remodeling through senescence-associated macrophage dysfunction
Source: JCI Insight. 2023 Apr 24;8(8):e145811. doi: 10.1172/jci.insight.145811 (PMC10243739; doi:10.1172/jci.insight.145811)
Supplement: Supplemental data [file jciinsight-8-145811-s122.pdf]

Osteopontin promotes age-related adipose tissue remodeling through senescence-associated macrophage dysfunction

Authors and affiliations
Daigo SAWAKI[1], Yanyan ZHANG[1], Amel MOHAMADI[1], Maria PINI[1], Zaineb MEZDARI[1], Larissa LIPSKAIA[1], Suzain NAUSHAD[1], Lucille LAMENDOUR[1], Dogus Murat ALTINTAS[1], Marielle BREAU[1], Hao LIANG[1], Maissa HALFAOUI[1], Thaïs DELMONT[1], Mathieu SURENAUD[1,2], Déborah ROUSSEAU[3], Takehiko YOSHIMITSU[4], Fawzia LOUACHE[5, 6], Serge ADNOT[1,7], Corneliu HENEGAR[1], Philippe GUAL[3], Gabor CZIBIK[1], Geneviève DERUMEAUX[1,7]

[1] INSERM U955, Université Paris-Est Créteil (UPEC), 8 rue du Général Sarrail, 94000 Créteil, France
[2] AP-HP Vaccine Research Institute (VRI), Créteil, France
[3] Université Côte d'Azur, INSERM U1065, C3M, Nice, France
[4] Laboratory of Synthetic Organic and Medicinal Chemistry, Graduate School of Medicine, Dentistry, and Pharmaceutical Sciences, Okayama University, Okayama, Japan
[5] Université Paris-Saclay, Inserm UMR-S-MD1197, Hôpital Paul Brousse, Villejuif, F-94800, France.
[6] Gustave Roussy Cancer Center, Villejuif, F-94805, France.
[7] AP-HP, Department of Physiology, Henri Mondor Hospital, FHU SENEC, Créteil, France
These authors contributed equally as 1st authors: Daigo SAWAKI, Yanyan ZHANG, Amel MOHAMADI, Maria PINI, and as senior authors: Gabor CZIBIK, Geneviève DERUMEAUX
Correspondence and requests for materials should be addressed to G.D. Mailing address: INSERM U955, Université Paris-Est Créteil (UPEC), 8 rue du Général Sarrail, 94000 Créteil, France; Phone: +33 (0)603 613517; email: genevieve.derumeaux@inserm.fr

Conflict of interest statement
The authors have declared that no conflict of interest exists.

Supplemental figure 1

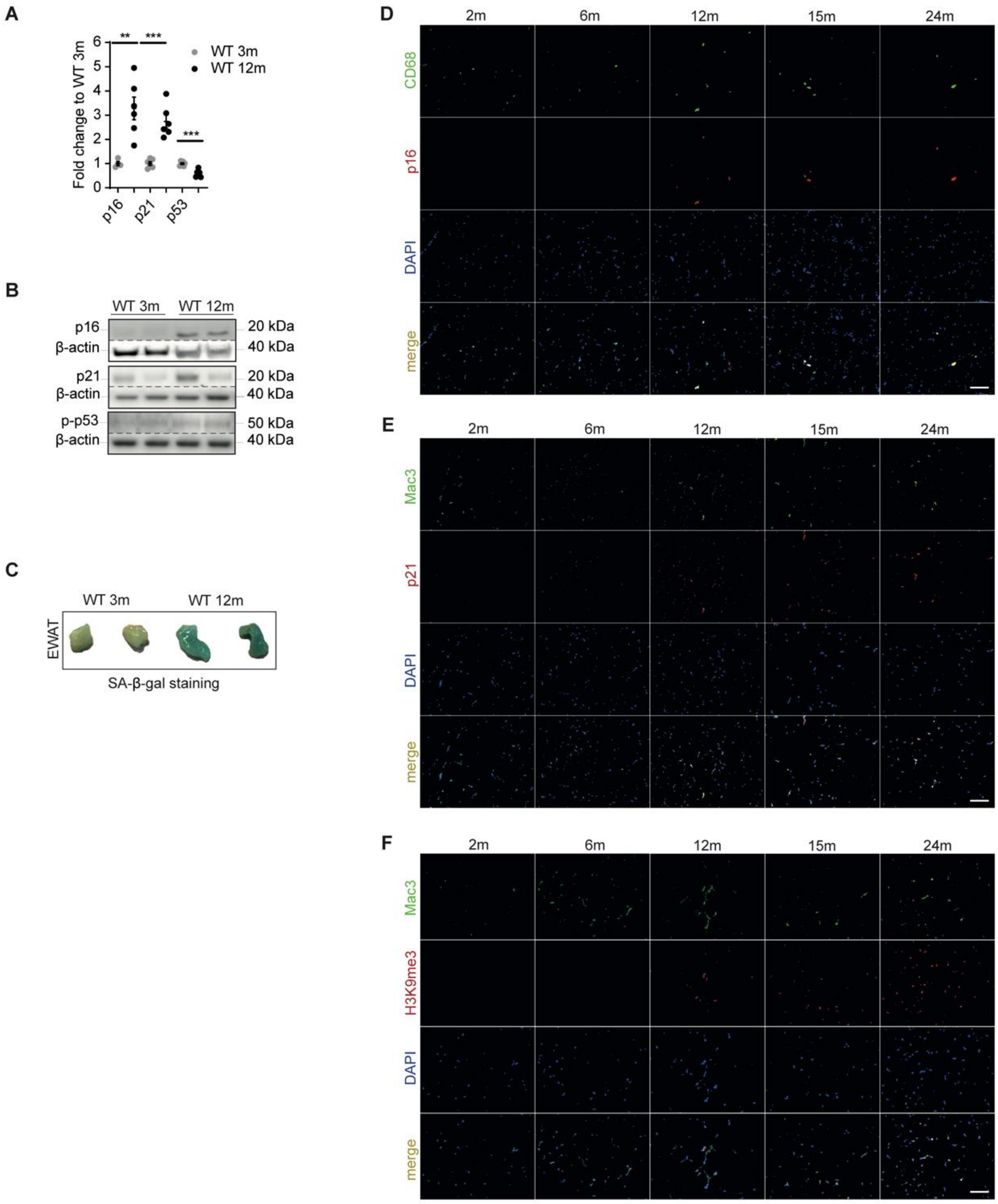

Supplemental figure 1 (continued)

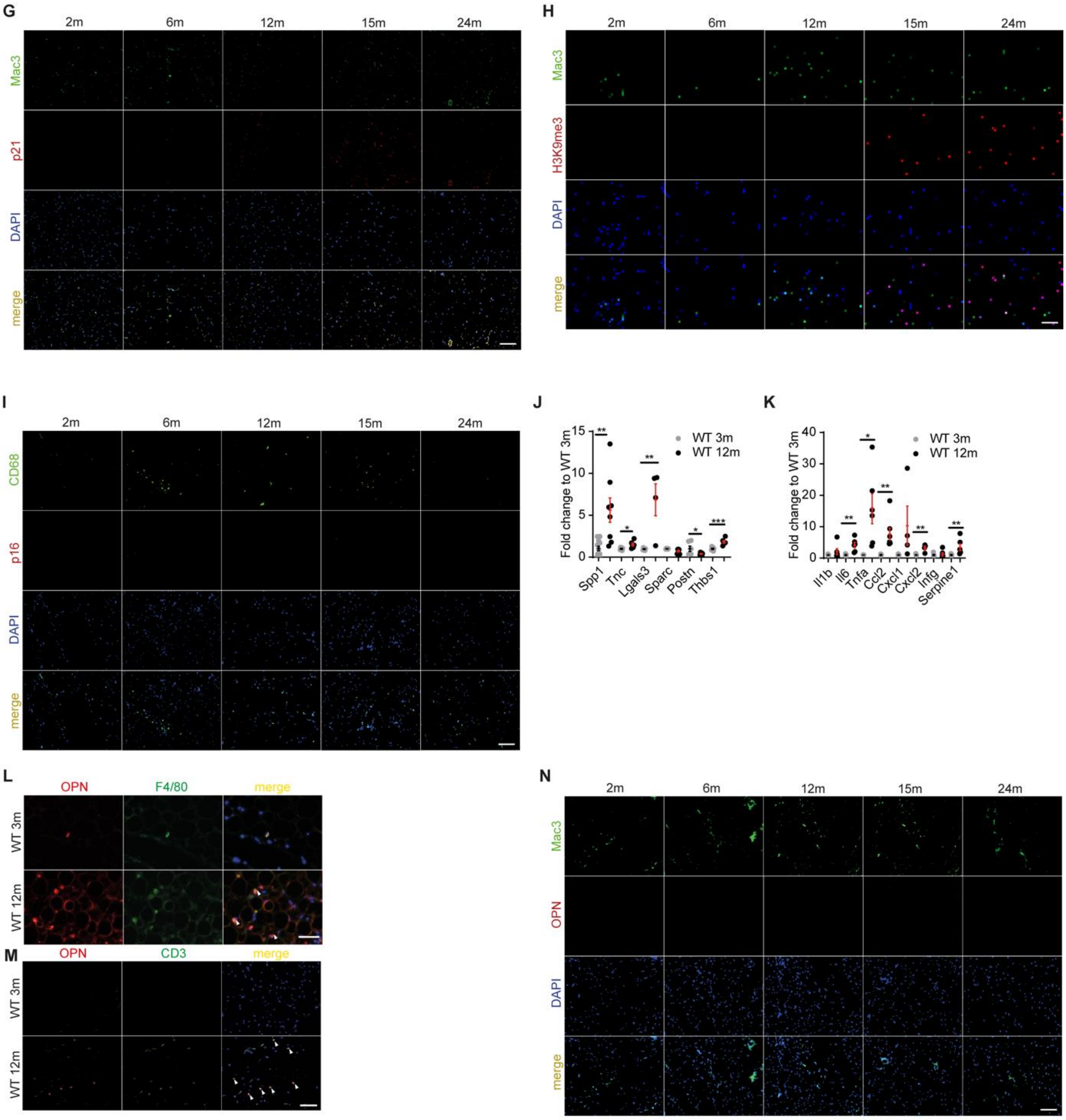

Supplemental figure 1 (continued)

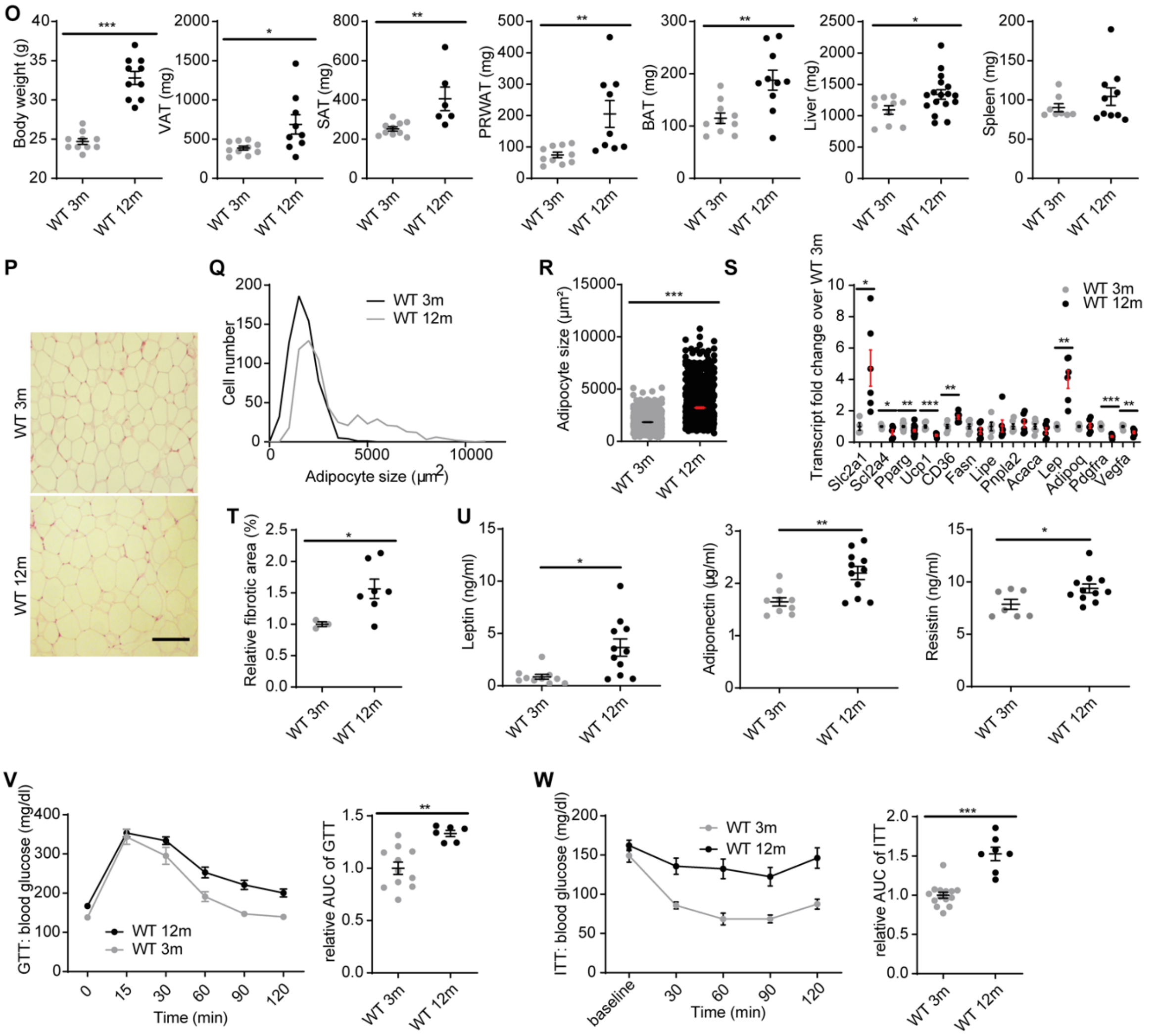

Supplemental Fig.1. Aging promotes accumulation of senescent cells in visceral adipose tissue. A qRT-PCR analysis of senescence markers (whole epididymal visceral adipose tissue: VAT) normalized to mean expression level of 3-month-old WT mice (n=6 mice/group). **B** Representative Western blot analysis using protein lysates derived from VAT of 3-month and 12-month WT mice. Separate loading controls (β-actin) are presented due to the non-contemporaneously run of the same lysates. **C** Representative SA-β-Gal staining (bluish green) of VAT from 3- and 12-month-old WT mice. **D-F** Representative immunofluorescent images of senescence markers (p16: **D**, p21: **E** & H3K9me3: **F**) counterstained with a macrophage marker (CD68 or Mac3) in VAT as indicated, scale bar=50 μm (n=3/group). **G-I** Representative immunofluorescent images of senescence markers (p21: **G**, H3K9me3: **H**, & p16: **I**) counterstained with a macrophage marker (CD68 or Mac3) in inguinal subcutaneous adipose tissue (SAT) as indicated, scale bar=50 μm (n=3/group). **J-K** qRT-PCR analysis (whole VAT) of matricellular proteins (**J**) and proinflammatory factors (**K**) normalized to the mean expression level of 3-month-old mice in animals as above (n=6 mice/group). **L-M** Representative immunofluorescence of VAT from 3-month and 12-month-old WT mice (OPN: red, F4/80: green (**L**), CD3: green (**M**), DAPI: blue), arrowheads indicate double-positive cells, scale bar=50 μm. **N** Representative immunofluorescent images of OPN counterstained with a macrophage marker (Mac3) in SAT, scale bar=50 μm (n=3/group). **O** Body weight and organ weight (VAT, SAT, perirenal (PRWAT) and brown adipose tissue (BAT), liver, spleen) derived from 3- and 12-month-old WT mice (n=10 mice/group). **P** Representative hematoxylin-eosin staining of VAT from 3- and 12-month-old mice, scale bar=25 μm. **Q-R** Distribution (**Q**) and difference (**R**) of adipocyte size in VAT, n=5 mice/group. **S** qRT-PCR

analysis (whole VAT) of metabolism-related gene expression levels normalized to the mean expression level of 3-month-old WT mice in VAT of 3- and 12-month-old WT mice (n=6 mice/group). **T** Relative fibrotic area by Sirius red staining in VAT of 3- and 12-month-old WT mice (n=3-7 mice/group). **U** Plasma adipokine levels by ELISA in 3- and 12-month-old WT mice (n=7-11 mice/group). **V** Glucose tolerance test (GTT) and **W** insulin tolerance test (ITT), temporal plot and area-under-curve (AUC) analysis (n=6-14 mice/group). Data is presented as original images (**B**-**I**, **L-N** & **P**) or individual values with mean ± SEM and analyzed with two-tailed, unpaired Student's t-test (**A**, **J**-**K**, **O, R**-**W**); ns: non-significant, *$p < 0.05$, **$p < 0.01$, ***$p < 0.001$, ****$p < 0.0001$.

Supplemental figure 2

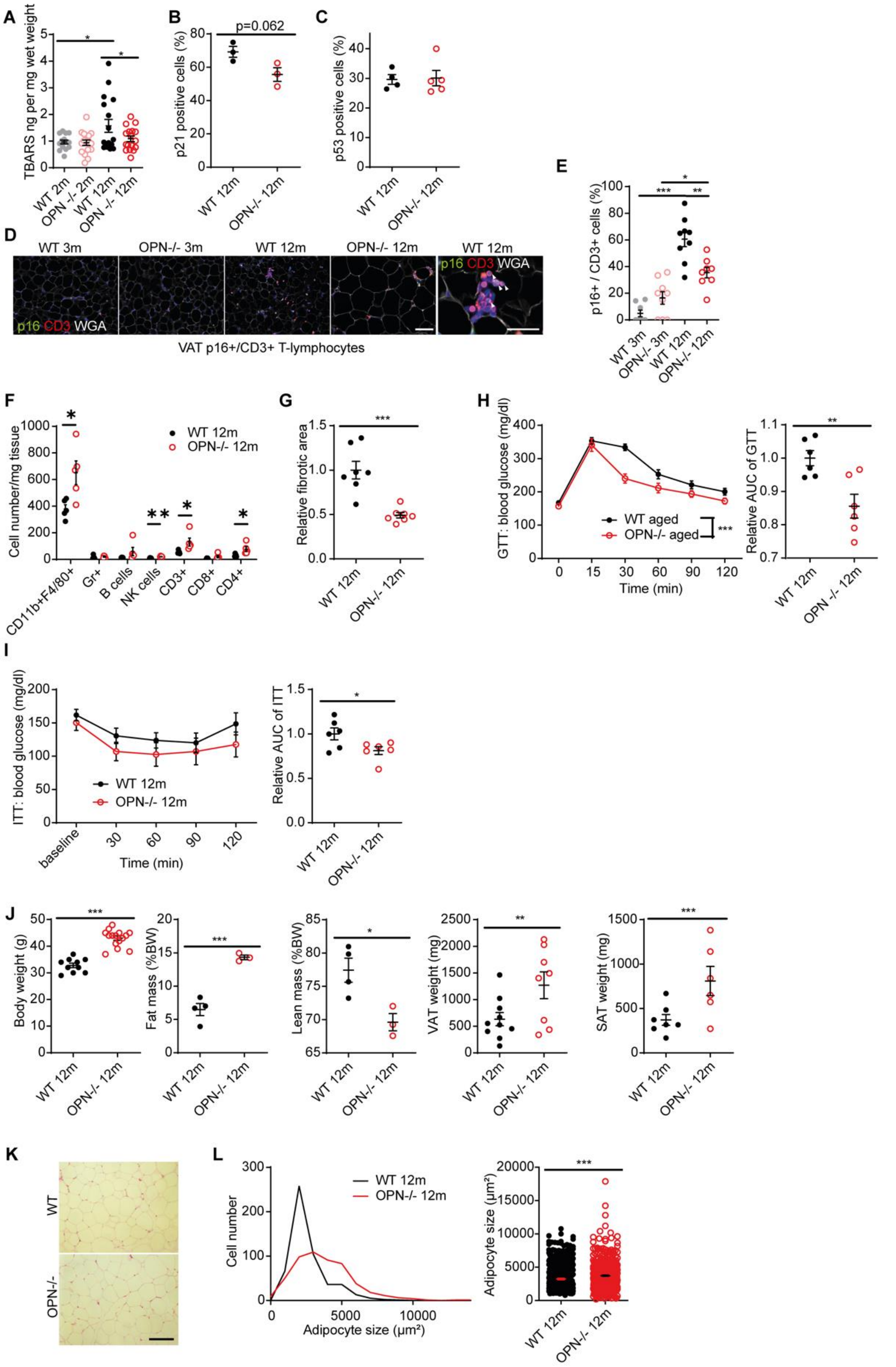

Supplemental figure 2 (continued)

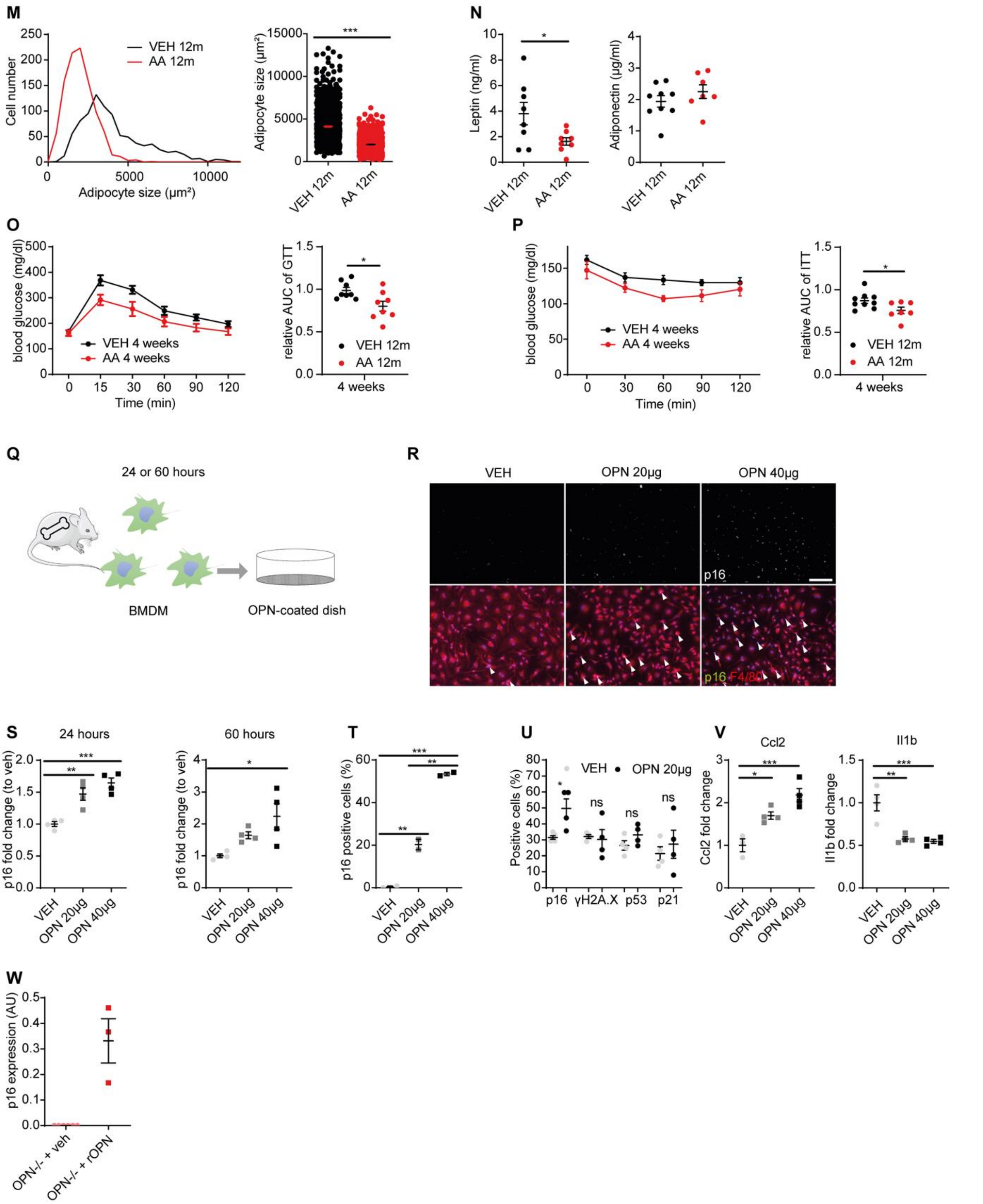

Supplemental Fig.2. Osteopontin is a critical regulator of adipose tissue and macrophage senescence. A Thiobarbiturate-reactive species (TBARS => malondialdehyde) in VAT derived from 2- and 12-month-old WT and OPN-/- mice. TBARS values were normalized to mg wet weight (n=14-17/group). **B-C** Quantification of p21- (**B**; n=3/group) and p53-positive (**C**; n=4-5/group) cells in VAT of 12-month-old WT and OPN-/- mice. **D-E** Representative immunofluorescence (**D**) and quantification (**E**; n=6-9/group) of p16/CD3 in VAT of 3- and 12-month-old WT and OPN-/-mice, scale bar=50 µm. **F** Immune cell numbers per milligram of wet weight (n=5/group)**. G** Relative fibrotic area by Sirius-red staining (n=7 mice/group). **H-I**

Glucose tolerance test (GTT; **H**) and insulin tolerance test (ITT; **I**) with 12-month-old WT and OPN-/- mice, temporal plot, and area under curve (AUC) analysis (n=6 mice/group). **J** Body weight (n=10 mice/group), body composition (n=3-4 mice/group) and fat weight (n=6-10 mice/group) in 12-month-old WT and OPN-/- mice. **K** Representative hematoxylin and eosin staining of VAT of 12-month-old WT and OPN-/- mice, scale bar=50 µm. **L** Distribution and difference of adipocyte size in VAT of 12-month-old WT and OPN-/- mice (n=5 mice/group). **M** Distribution and difference of adipocyte size in VAT of vehicle (VEH)- or Agelastatin A (AA)-treated 12-month-old WT mice (n=8 mice/group). **N** Plasma adipokine levels by ELISA (n=7-9 mice/group). **O**-**P** GTT (**O**) and ITT (**P**), temporal plot and area under curve (AUC) analysis in vehicle- or AA-treated 12-month-old WT mice (n=8 mice/group). **Q** Protocol of *in vitro* stimulation of bone marrow-derived macrophage (BMDM) with OPN. **R** Representative immunofluorescence of p16 (white spots) of WT BMDM treated with vehicle or OPN-coated dish at indicated dose of OPN (upper row), and representative merged images of immunofluorescence (lower row, p16: green, F4/80: red, DAPI: blue), arrowheads indicate p16 and F4/80 double-positive cells, scale bar=50 µm. **S** qRT-PCR analysis of *Cdkn2a* (p16) gene expression in vehicle- or OPN-treated WT BMDM. **T** p16-positive cells in percentage of total cell number (n=3 independent experiments). **U** Quantification of p16, γH2A.X, p53 and p21 positive WT BMDM treated with vehicle or OPN (n=4/condition). **V** qRT-PCR analysis of *Ccl2* and *Il1b* gene expression after vehicle or OPN treatment (20 or 40 µg for 60 hours) normalized to the mean expression of vehicle-treated BMDM (n=4/group). **W** qRT-PCR analysis of *p16* gene expression in OPN-/- BMDM after vehicle or OPN treatment (20 µg for 24 hours, n=3-6/group; AU: arbitrary units). Data is presented as original images (**D**, **K**, **R**) or individual values with mean ± SEM and analyzed with two-tailed, unpaired Student's t-test (**B-C**, **F-P,** **U**, **W**) or one-way ANOVA with Bonferroni post-hoc test (**A**, **E,** **S**-**T**, **V**); ns: non-significant, *$p < 0.05$, **$p < 0.01$, ***$p < 0.001$, ****$p < 0.0001$.

Supplemental figure 3

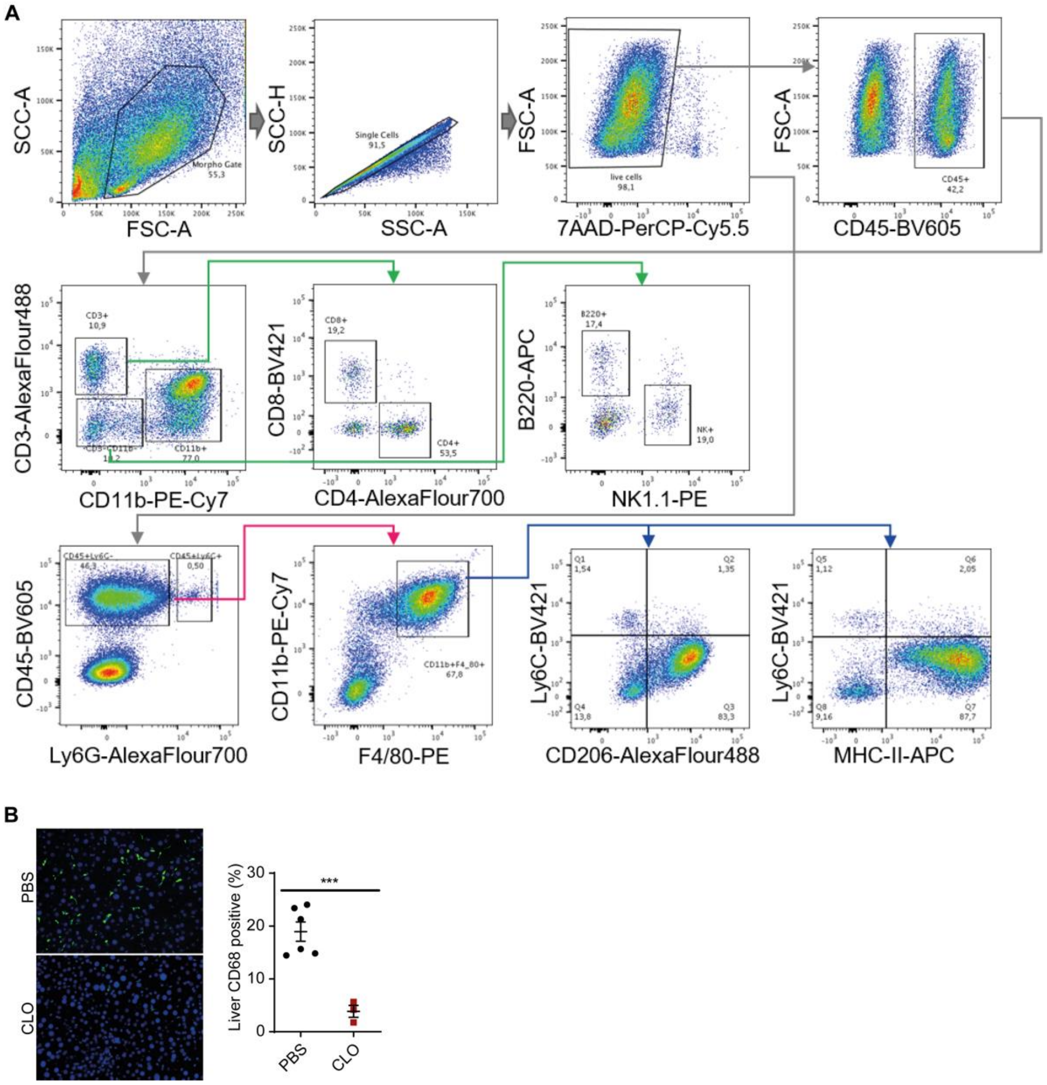

Supplemental Fig.3. Adipose tissue macrophages promote age-dependent VAT and metabolic abnormalities. A Flow cytometry gating strategy for separating immune cell populations; CD11b+F4/80+: macrophages, Ly6G+: neutrophiles, B220+: B-cells, NK1.1+: NK cells, CD3+CD4+ or CD3+CD8+: T-cells. Flow cytometry gating strategy for separating macrophage/monocyte subpopulation; CD45+Ly6G-CD11b+F4/80+ macrophages were further separated to Ly6C/CD206 and Ly6C/MHC-II subpopulations. **B** Representative CD68 immunofluorescence of livers from 12-month-old WT mice treated with PBS or clodronate (CD68: green, DAPI: blue), and quantification of CD68-positive cells (green; n=4-6 mice/group) in percentage of total cell number (DAPI), scale bar=50 µm. Data is presented as original images (**A**-**B**) or individual values (**B**) with mean ± SEM and analyzed with two-tailed, unpaired Student's t-test (**B**); ns: non-significant, *p < 0.05, **p < 0.01, ***p < 0.001, ****p < 0.0001.

Supplemental figure 4

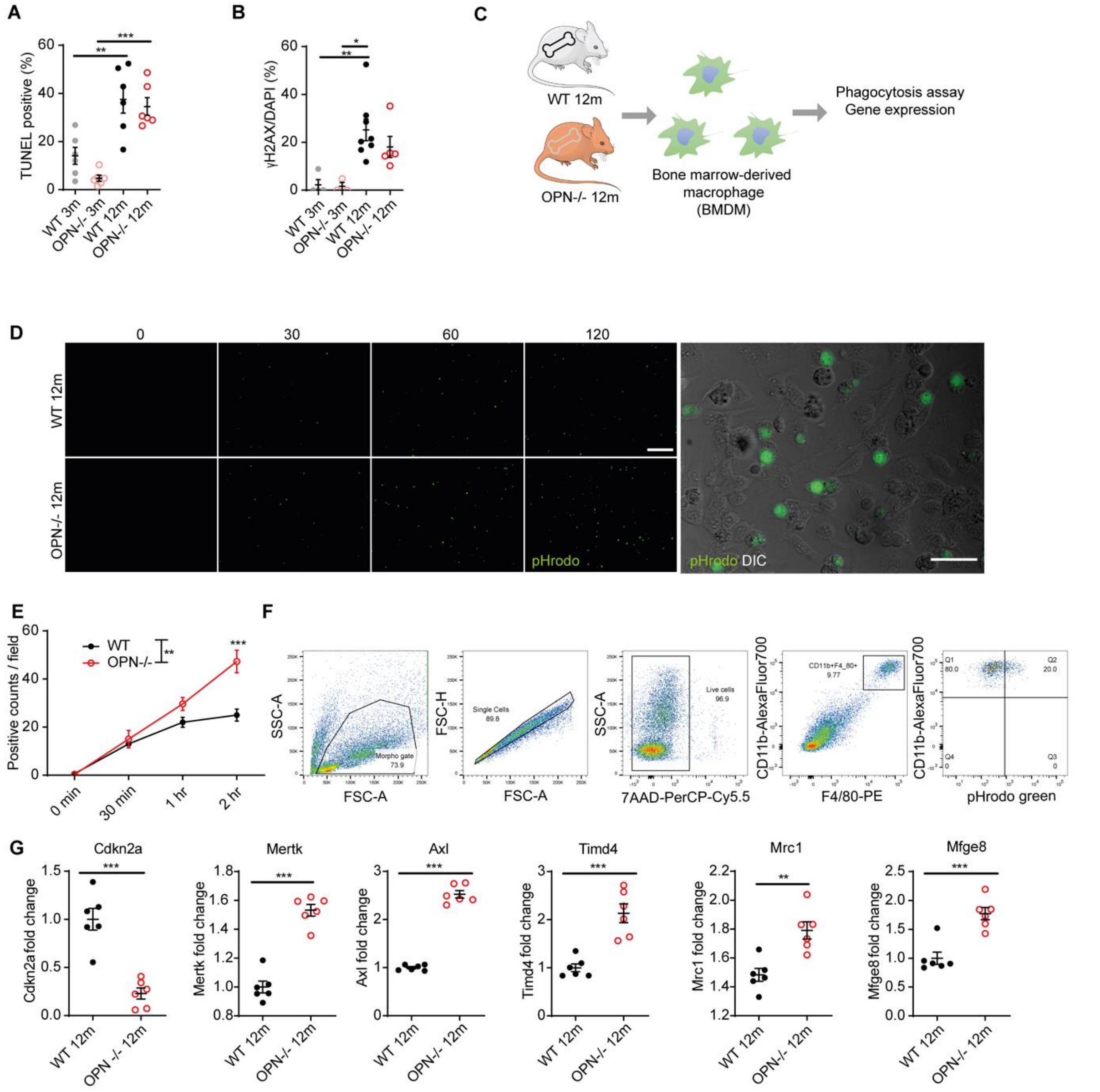

Supplemental Fig.4. Role of OPN in age-related ATM dysfunction. A-B Quantification of TUNEL- (**A**) and γH2A.X-positive (**B**) cells in percentage of total cell number in VAT of 3- and 12-month-old WT and OPN-/- mice (n=5-8 mice/group). **C** Protocol of *in vitro* BMDM experiments. **D** Representative chronological fluorescent microscopic images using BMDM and pHrodo-labeled human leukemia cells (green) and a magnified picture with differential interference contrast image (DIC), scale bar=50 μm. **E** Microscopic quantification of fluorescent-positive BMDM incubated with pHrodo-labeled human leukemia cells at indicated timepoints (n=3 independent experiments). **F** Flow cytometry gating strategy of phagocytosis analysis using BMDM and pHrodo-labeled human leukemia cells. **G** qRT-PCR analysis of senescence and efferocytosis-related gene expression in BMDM derived from 12-month-old WT and OPN -/- mice (n=6 mice/group). Data is presented as original images (**D**, **F**) or individual values with mean ± SEM and analyzed with two-tailed, unpaired Student's t-test (**G**), one-way ANOVA with Bonferroni post-hoc test (**A**-**B**) or 2-way ANOVA (**E**); ns: non-significant, **p* < 0.05, ***p* < 0.01, ****p* < 0.001, *****p* < 0.0001.

Supplemental figure 5

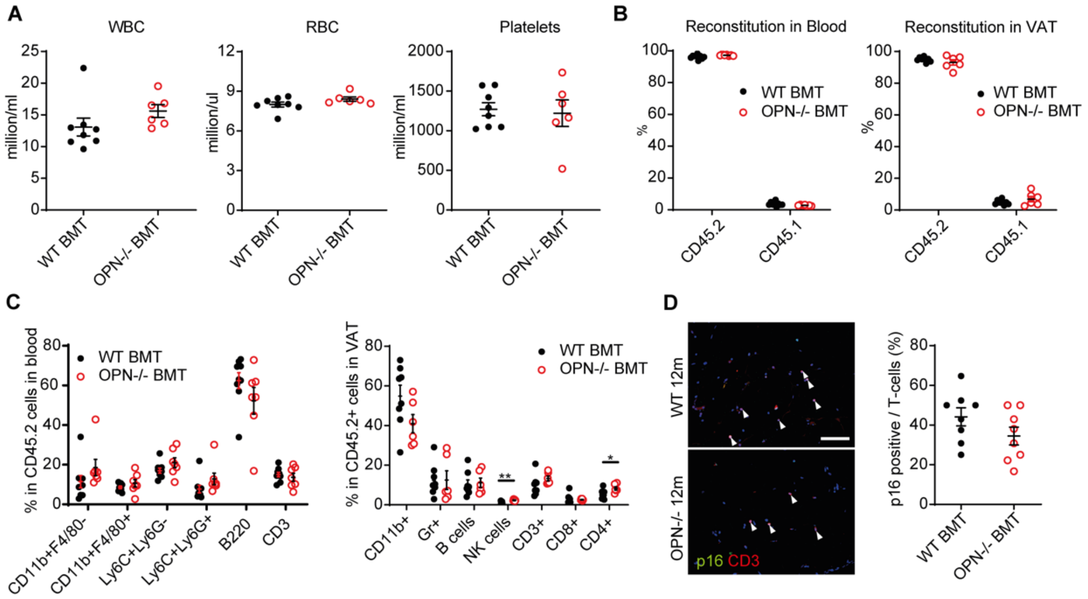

Supplemental Fig.5. Transplantation of OPN-/- bone marrow rejuvenates visceral adipose tissue and improves metabolic function during aging. 2-month-old WT mice received bone marrow transplantation (BMT) from WT and OPN-/- donors and were sacrificed 12 months later (2+12m WT & OPN-/-). **A-B** Peripheral blood count for leukocytes (WBC; **A**), red blood cells (RBC; **B**), and platelets and reconstitution in blood (quantification of CD45.1 or CD45.2 (chimerism) percentage in blood leukocyte populations; **B**) at the time of sacrifice (12 months after BMT) by flow cytometry (n=7-9 mice/group). **C** Quantification of blood and VAT leukocyte subpopulations defined by indicated surface markers. (n=6 mice/group). **D** Representative p16/CD3 immunofluorescence of VAT from WT or OPN-/- BMT mice (p16: green, CD3: red, DAPI: blue), with quantification of p16-positive cells in the percentage of CD3-positive cells (n=4 mice/group). Arrowheads indicate double-positive cells, scale bar=50 μm. Data is presented as original images (**D**) or individual values with mean ± SEM and analyzed with two-tailed, unpaired Student's t-test (**A-D**); ns: non-significant, *$p < 0.05$, **$p < 0.01$, ***$p < 0.001$, ****$p < 0.0001$.

Supplemental table: Taqman assays and antibodies

Taqman assays

Target gene	Taqman assay ID	Target gene	Taqman assay ID
Cdkn2a	Mm00494449_m1	***Acaca***	Mm01304257_m1
Cdkn1a	Mm04205640_g1	***Pparg***	Mm00440940_m1
Tp53	Mm01731290_g1	***Pnpla2***	Mm00503040_m1
Mfge8	Mm00500549_m1	***Adipoq***	Mm00456425_m1
Timd4	Mm00724709_m1	***Il6***	Mm00446190_m1
Mertk	Mm00434920_m1	***Tnf***	Mm00443258_m1
Mrc1	Mm01329362_m1	***Mif***	Mm01611157-g1
Spp1	Mm00436767_m1	***Serpine1***	Mm00435858_m1
Thbs1	Mm01335418_m1	***Vegfa***	Mm01283063_m1
Sparc	Mm00486332_m1	***Il12b***	Mm01288989
Lgals3	Mm00802901_m1	***Cxcl2***	Mm00436450_m1
Tnc	Mm00495662_m1	***Ccl2***	Mm00441242_m1
Postn	Mm00450111_m1	***Cxcl1***	Mm04207460_m1
Pdgfra	Mm01205760_m1	***Ihnbb***	Mm03023992_m1
Lep	Mm00434759_m1	***Tgfb1***	Mm01178820_m1
Cd36	Mm00432403_m1	***Il12a***	Mm00434169_m1
Slc2a1	Mm00441480	***Cxcl5***	Mm00436451_g1
Ucp1	Mm01244861_m1	***Csf2***	Mm01290062
Pgc1a	Mm01208835_m1	***Csf1***	Mm00432686
Slc2a4	Mm00436615_m1	***Il1b***	Mm00434228_m1
Plin1	Mm00558672_m1	***Infg***	Mm01168134_m1
Fasn	Mm00662319_m1	***Actb***	Mm02619580_g1
Lipe	Mm00495359_m1	***Axl***	Mm00437221_m1

Antibodies used for immunohistochemistry and Western blot analysis

Target protein	Clone/product number	Company	Application/concentration
**H3K9me3**	ab176916	abcam	IF: 200^{-1}
**p16**	2D9A12/ab54210	abcam	IF: 200^{-1}
**p16**	polyclonal/250804	Abbiotec	IF: 200^{-1}, WB: 200^{-1}
**p21**	HUGO291	EuroMabNet	IF: 200^{-1}, WB: 500^{-1}
**p21**	ab188224	abcam	IF: 200^{-1}
**p53**	polyclonal/ab31333	abcam	IF: 200^{-1}
**phospho-p53 (Ser15)**	polyclonal/9284	Cell Signaling	WB: 1000^{-1}
**Osteopontin**	polyclonal/ab8448	abcam	IF: 200^{-1}, WB: 500^{-1}
**F4/80**	A3-1/MCA497GA	Serotec	IF: 200^{-1}
**β-actin**	AC-15/ab49900	abcam	WB: 5000^{-1}
**Wheat germ agglutinin**	W11261	Invitrogen	IF: 200^{-1}
**Mac3**	BD 550292	BD Biosciences	IF: 200^{-1}
**CD68**	polyclonal/ab125212	abcam	IF: 200^{-1}

**CD3**	polyclonal/ab5690	abcam	IF: 200^{-1}
**CD45**	30-F11/550539	BD Pharmingen	IF: 200^{-1}
**GammaH2A.X(phosphoS140)**	3F2/ab22551	abcam	IF: 200^{-1}
**Cleaved caspase3**	5A1E/9664	Cell Signaling	IF: 200^{-1}

Antibodies and reagents used for flow cytometry

Antibodies or Reagents	Clone	Isotype	Source	Identifier
**Brilliant Violet 605™ anti-mouse CD45**	30-F11	Rat IgG2b, k	Biolegend	103155
**APC/Cyanine7 anti-mouse CD45.2**	104	Mouse IgG2a, κ	Biolegend	109823
**Brilliant Violet 605™ anti-mouse CD45.1**	A20	Mouse IgG2a, κ	Biolegend	110737
**PE/Cy7 anti-mouse/human CD11b**	M1/70	Rat IgG2b, k	Biolegend	101216
**PE anti-mouse F4/80**	BM8	Rat IgG2a, k	Biolegend	123110
**Alexa Fluor® 700 anti-mouse Ly-6G**	1A8	Rat IgG2a, k	Biolegend	127622
**APC anti-mouse I-Ab**	AF6-120.1	Rat IgG2a, k	Biolegend	116418
**Brilliant Violet 421™ anti-mouse Ly-6C**	HK1.4	Rat IgG2c, k	Biolegend	128031
**FITC anti-mouse CD206**	MR5D3	Rat IgG2a, k	Thermofisher Scientific	MA5-16870
**Brilliant Violet 650™ anti-mouse CD206 (MMR)**	C068C2	Rat IgG2a, k	Biolegend	141723
**Brilliant Violet 785™ anti-mouse CD11c**	N418	A. Hamster IgG	Biolegend	117335
**APC/Cyanine7 anti-mouse CD3**	17A2	Rat IgG2b, k	Biolegend	100222
**FITC anti-mouse CD3ε**	145-2C11	A. Hamster IgG	Biolegend	100305
**Alexa Fluor® 700 anti-mouse CD4**	RM4-4	Rat IgG2b, k	Biolegend	116021
**Brilliant Violet 421™ anti-mouse CD8a**	53-6.7	Rat IgG2a, k	Biolegend	100737
**APC anti-mouse/human CD45R/B220**	RA3-6B2	Rat IgG2a, k	Biolegend	103212
**PE anti-mouse NK-1.1**	PK136	Rat IgG2a, k	Biolegend	108707
**AF700 anti-mouse/Rat CD29**	HMβ1-1	A. Hamster	Biolegend	102218
**BV421 anti mouse/human CD44**	IM7	Rat IgG2b, k	Biolegend	103039
**FITC anti-mouse CD49d**	R1-2	Rat IgG2b, k	Biolegend	103605
**PE anti-mouse/rat CD49e**	HMα5-1	Armenian Hamster IgG	Biolegend	103905
**PE anti-mouse CD51 antibody**	RMV-7	Rat IgG1, κ	Biolegend	104105
**FITC anti mouse/rat CD61**	2C9.G2	Armenian	Biolegend	104305

**antibody**	(HMß3-1)	Hamster IgG		
**PE Rat IgG1, κ Isotype Ctrl**	RTK2071		Biolegend	400408
**PE/Cy7 Rat IgG2b, κ Isotype Ctrl**	RTK4530		Biolegend	400617
**FITC Rat IgG2a, κ Isotype Ctrl**	RTK2758		Biolegend	400505
**APC Rat IgG2b, κ Isotype Ctrl**	RTK4530		Biolegend	400611
**APC/Cy7 Rat IgG2a, κ Isotype Ctrl**	RTK2758		Biolegend	400523
**Brilliant Violet 421™ Isotype Ctrl**	TRK2758	Rat IgG2a, κ	Biolegend	400535
**Brilliant Violet 605™ Isotype Ctrl**	RTK4530	Rat IgG2b, κ	Biolegend	400649
**Brillant violet 650 isotype control**	RTK2758	Rat IgG2a, k	Biolegend	400541
**Rat IgG2b Alexa Fluor® 700-conjugated**	141945		R&D	IC013N
**TruStain FcX™ (anti-mouse CD16/32)**	93		Biolegend	101320
**7-aminoactinomycin D (7-AAD)**			Invitrogen	A1310
**OneComp eBeads**			Invitrogen	01-1111-42
